# VDR and deubiquitination control neuronal oxidative stress and microglial inflammation in Parkinson’s disease

**DOI:** 10.1038/s41420-024-01912-9

**Published:** 2024-03-21

**Authors:** Zihui Zheng, Miao Chen, Shengliang Feng, Huanhuan Zhao, Tiange Qu, Xudong Zhao, Qinli Ruan, Lei Li, Jun Guo

**Affiliations:** 1 School of Medicine & Holistic Integrative Medicine, Nanjing University of Chinese Medicine, Nanjing, 210023 Jiangsu P. R. China; 2 Department of General Practice, Affiliated Hospital of Xuzhou Medical University, 99 Huaihai West Road, Xuzhou, 221002 Jiangsu P. R. China

**Keywords:** Cellular neuroscience, Diseases of the nervous system

## Abstract

Close correlation between vitamin D (VitD) deficiency and Parkinson’s Disease (PD) risk, VitD as an adjuvant treatment promising to improve PD progression. However, VitD excessive intake could induce hypercalcemia and renal damage. Therefore, upregulation of vitD receptor (VDR) is considered a compensatory strategy to overcome VitD insufficiency and alleviate PD symptoms. In this study, we discovered that VDR played antioxidative roles in dopaminergic neurons by decreasing reactive oxygen species (ROS) and maintaining mitochondrial membrane potential. Further, we newly identified VDR downstream events in *C. elegans*, including glutathione S-transferase (*gst*) and forkhead box transcription factor class O (*daf-16*) mediated oxidative stress resistance. VDR upregulation also mitigated microglial activation through inhibition of NLRP3/caspase-1-mediated inflammation and membrane permeabilization. These findings highlight the multifaceted protective effects of VDR in both neurons and microglia against the development of PD. Importantly, we discovered a novel deubiquitinase DUB3, whose N-terminal catalytic domain interacted with the C-terminal ligand-binding domain of VDR to reduce VDR ubiquitination. Identification of DUB3 as an essential player in the deubiquitinating mechanism of VDR provides valuable insights into VDR regulation and its potential as a therapeutic target for PD.

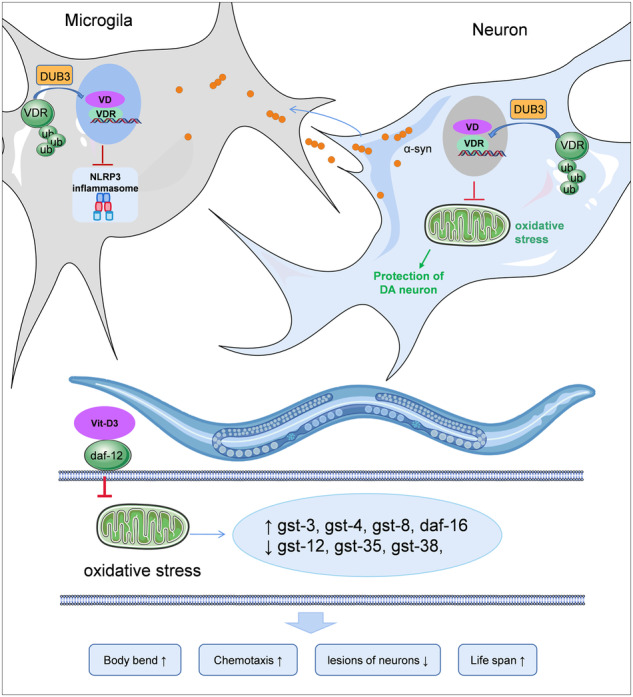

## Introduction

Parkinson’s disease (PD) is a prevalent neurodegenerative disorder, characterized by motor deficits and misfolded α-synuclein (α-syn) accumulation, and dopaminergic neuronal death in the substantia nigra. Multiple signaling pathways, including mitochondrial dysfunction, abnormal protein misfolding, and neuroinflammation, cooperate to induce dopaminergic neuronopathy, being in the vicious cycle of PD pathogenesis [1–3]. These complex mechanisms highlight the need to identify specific target that can effectively modulate the multiple downstream events associated with PD and potentially slow down disease progression.

Clinical studies find that hypovitaminosis D is closely related to PD. A high prevalence of vitamin D (VitD) deficiency (25-OH-D concentrations lower than 50 nmol/L) and insufficiency (concentrations between 50 and 75 nmol/L) in PD patients has been reported [4–6]. PD patients with VitD deficiency present wider brain regions with the changed fraction amplitude of low-frequency fluctuation and spontaneous neuronal activity [7]. The low VitD doses are closely correlated with the PD risk and severity of motor disorders [8–10]. In vitro cell lines and in vivo rodent research find that VitD facilitates the expression of tyrosine hydroxylase (TH) involved in developing the dopamine system, alleviating the damage of dopaminergic neurons and inflammation [11–13]. Some clinical trials and meta-analyses report VitD supplementation in PD patients can improve motor symptoms, whereas other research provides conflicting findings [14–17], suggesting the necessary investigations of VitD interventional mechanism in the PD progression.

VitD signal activation depends on vitamin D receptor (VDR). VDR polymorphisms, particularly FokI allele, have been reported to be associated with PD risk and severity [18–20]. VitD facilitates VDR and retinoic X receptor (RXR) interaction to form complex, which is internalized into the nucleus and binds with the VitD response elements (VDREs), able to regulate the transcription of multiple downstream genes [21]. Notably, excessive intake of VitD could lead to hypercalcemia and renal damage [22, 23], and its safety for PD treatment is limited. Upregulation of VDR expression and function is promising to compensate VitD deficiency. VDR, abundantly expressed in SN neurons and glial cells, is crucial for the development of dopaminergic neurons and cognitive function [24]. In pathological conditions, VDR can regulate the inflammatory response via interacting with NLRP3 inflammasome. Therefore, targeting the VitD-VDR pathway is an effective strategy to alleviate the pathological progression of PD; but the prerequisite is to investigate the entry point for regulating VDR.

Recent studies have demonstrated that VDR is regulated by ubiquitination, and E3 ubiquitin ligase MDM2 mediates the ubiquitination degradation of VDR in lung adenocarcinoma cells [25]. Deubiquitinating enzymes (DUBs) are involved in the ubiquitin-proteasome system by removing the polyubiquitin chain from the substrate protein [26]. This raises a question: what roles does VDR deubiquitinating regulation play in the PD process? The present study identifies particular deubiquitinase to prevent VDR degradation and up-regulate VDR neuroprotective function, further synergistically modulating multiple downstream signaling pathways in PD pathogenesis, which provides a new target and direction for the PD treatment.

## Results

### VDR rescues rotenone-induced ROS in neuron

**Fig. 1:**
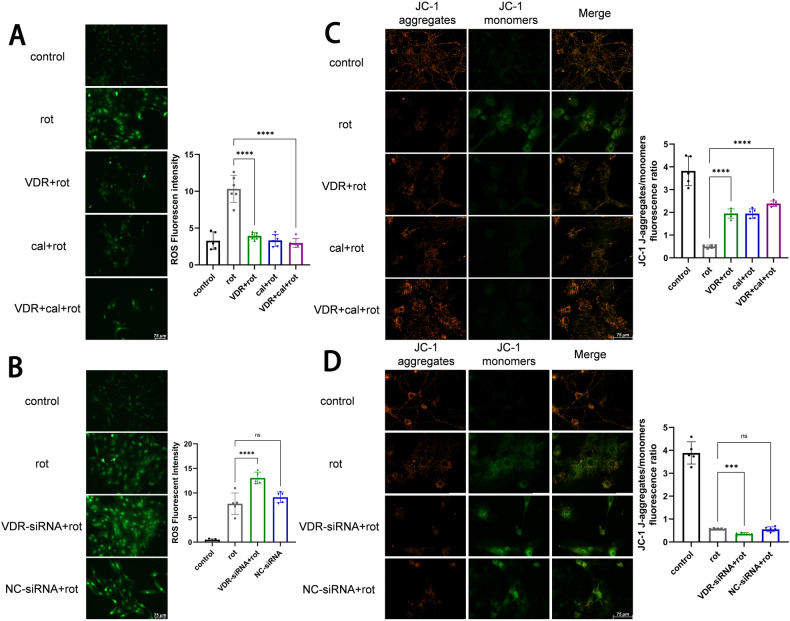
VDR alleviates rotenone-induced mitochondrial dysfunction in primary neurons. **A**, **B** Fluorescence images of primary neurons (co-)treated with rotenone (rot, 2 μM, 24 hour), calcitriol (cal, 100 nM, 24 hours) in VDR overexpression or VDR-siRNA knockdown, ROS was labeled with a DCFH-DA probe. The bar charts represent ROS fluorescence intensity analysis. Scale bar, 75 µm. **C**, **D** Fluorescence images of JC-1 (JC-1 aggregate, red; JC-1 monomer, green) stained primary neurons to assess the MMP. Scale bar, 75 µm. Data present as the mean ± SEM; *n* ≥ 5 biologically independent replicates. ***P < 0.001 and ****P < 0.0001, ns, no significant difference.

VDR is widely expressed in the central nervous system (CNS). Bound with calcitriol, VDR is internalized into the nucleus and acts as transcription factor, involved in the development of dopaminergic neurons [21]. Rotenone can induce PD-like etiopathogenesis in rodent models, including dopaminergic neuronal impairment and neurodegeneration [27, 28]. We used rotenone treating with the primary neuron and dopaminergic neuron cell line MN9D. VDR expression was reduced in the rotenone group (Fig. S1A, Fig. S8). Mitochondrial dysfunction and oxidative stress involved in PD pathogenesis. Rotenone inhibits the mitochondrial complex I, leading to an increase in oxidative stress [28, 29]. VDR overexpression significantly reversed the rotenone-induced ROS, whose level trends were similar to the calcitriol and calcitriol-VDR cotreatments (Fig. 1A; Fig. S2A). These suggest VDR gain-function achieved the VitD-treated effects. Inversely, VDR-siRNA knockdown further elevated the rotenone-induced ROS levels (Fig. 1B; Fig. S2B; Fig. S3A). In addition, mitochondrial membrane potential (MMP) was measured by the JC-1 probe [30]. Results presented rotenone-induced JC-1 monomers were recovered to aggregates after VDR overexpression, representing the increased MMP (Fig. 1C; Fig. S2C); whereas the further lost MMP appeared after VDR knockdown (Fig. 1D; Fig. S2D). Therefore, VDR can suppress rotenone-induced oxidative stress and MMP disequilibrium, playing protective roles in neurons.

### VDR antagonizes the NLRP3-induced microglial activation

**Fig. 2:**
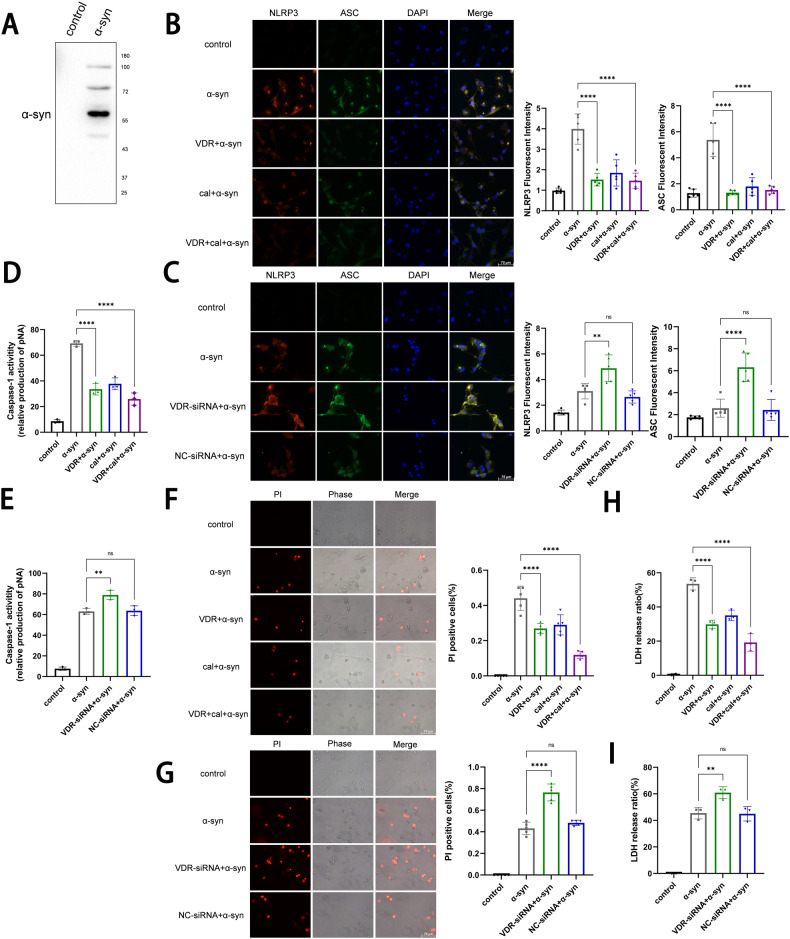
VDR inhibits α-syn-induced primary microglial inflammatory response. **A** Total protein isolated by ice acetone precipitation and centrifugation from the total medium of MN9D (dopaminergic neuron cell line) treated with α‐syn overexpression for 24 h was subjected to immunoblotting using α‐syn antibodies. **B**, **C** Fluorescent images showing primary microglia treated with the medium of α‐syn-overexpressed MN9D culture followed by calcitriol (100 nM, 24 hours) treatment, VDR overexpression, and VDR-siRNA knockdown in primary microglia, labeled with DAPI (blue), NLRP3 (red) and ASC (green) as well as the merge (yellow). The bar charts represent the quantification of NLRP3 and ASC fluorescence intensity analysis. Scale bar, 75 µm. **D**, **E** Caspase-1 activity of primary microglia under the different treatments. **F**, **G** Representative images of primary microglia subjected to different treatments. Cell membrane permeabilization was monitored by PI uptake (red fluorescence). Bar charts represent the percentage of PI-positive cells. Scale bar, 75 μm. **H**, **I** LDH release detected by the kit to verify cell membrane integrity. Data present as the mean ± SEM; *n* ≥ 3 biologically independent replicates. **P < 0.01 and ****P < 0.0001, ns, no significant difference.

PD clinical studies find the activated microglia clustered near Lewy bodies (LBs), accompanied by increased levels of pro-inflammatory cytokines. Released α-syn from neurons being uptaken by microglia, stimulates NLRP3 (NOD-, LRR- and pyrin domain-containing 3) inflammasomes, which further activates caspase-1 and downstream inflammatory cytokines [3]. The primary microglia and BV2 cell line were individually exposed in the medium cultured with α-syn-overexpressed MN9D dopaminergic neurons. Western blotting detected α-syn aggregates in the medium, released from the α-syn-overexpressed MN9D cells (Fig. 2A, Fig. S8). In the α-syn-induced microglia model, VDR expression was reduced (Fig. S1B, Fig. S8), and NLRP3-induced inflammation and membrane permeabilization occurred, showing the higher NLRP3/ASC expression, caspase-1 activity, percentage of propidium iodide (PI) positive cells and LDH release (Fig. 2B–I; Fig. S4A–D). This model was used to investigate VDR function in microglia. We found VDR overexpression or VDR-calcitriol cotreatments significantly inhibited NLRP3/ASC expression, caspase-1 activity, the IL-1β, IL-18 transcript levels, and cell permeabilization (Fig. 2B, D, F, H; Fig. S4A, B, E–H; S5A, B). However, VDR-siRNA further enhanced the inflammatory effects (Fig. 2C, E, G, I; Fig. S3B; Fig. S4C, D; S5C, D), suggesting VDR against the NLRP3-induced microglial activation.

### VitD-VDR protects from dopaminergic neuronal death, α-syn and motor deficits in the PD *C. elegans* model

**Fig. 3:**
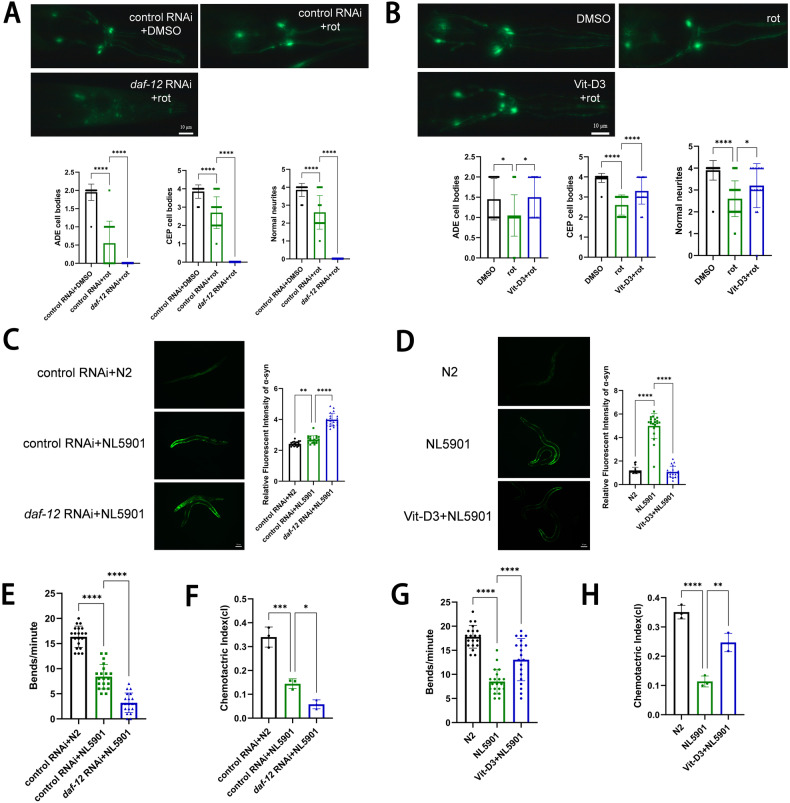
VitD-VDR protects against dopaminergic neuronal death in rotenone-induced *C. elegans* model, and alleviates motor dysfunction in α-syn transgenic *C. elegans* model. **A**, **B** Representative images and quantification of CEP and ADE dopaminergic cell bodies and neurites labeled with GFP on the 8th day with *daf-12* (VDR ortholog in *C. elegans*) knockdown (**A**) or Vitamin D3 treatment (**B**) in rotenone-exposed UA57 strain (fluorescent phenotyping of dopaminergic neurons). Scale bar, 10 μm. *n* = 20 nematodes. **C**, **D** Representative images and fluorescence intensity quantification of α-syn accumulation on Day 8 after treatment with *daf-12* knockdown (**C**) or Vitamin D3 (**D**) in NL5901 α-syn transgenic strain, respectively. N2 Strain Line serves as the wild-type control. Scale bar, 50 μm. *n* = 20. **E**, **G** Body bends of NL5901 nematodes treated with *daf-12* knockdown (**E**) or Vitamin D3 (**G**) with 6 μM rotenone exposure of eight days. *n* = 30 nematodes. **F**, **H** Chemotactic index of NL5901 strain in different treatments. *n* = 3 replicates (around 100 nematodes/each). Data present as the mean ± SEM. *P < 0.05, **P < 0.01, ***P < 0.001, and ****P < 0.0001.

*Caenorhabditis elegans* (*C. elegans*) contains 302 neurons with well-mapped electron micrographs, thereby widely used to study neurodegenerative diseases, such as PD and Alzheimer’s disease [31]. Rotenone induces cytotoxic damage and morphological destruction of neuron by elevating ROS, resulting in neurodegenerative degeneration and behavioral defects in nematode, established as the PD *C. elegans* model [32, 33]. In this study, nematodes were continuously exposed to rotenone till the senile stage (day 8 adult nematodes). In the *C. elegans* Line OH14589 [34], which expresses *daf-12::GFP::3×Flag* with endogenous *daf-12* promoter, the *daf-12* (VDR ortholog in *C. elegans*) protein levels were decreased after rotenone treatment (Fig. S6B), consistent with the PD cell model. We performed adult-specific RNA interference (RNAi) mediated *daf-12* knockdown in *C. elegans* to avoid embryo/larval lethality, and confirmed *daf-12* RNAi efficacy (Fig. S6C). *C. elegans* carries three pairs of anterior dopaminergic neurons, including two pairs of cephalic (CEP) and one pair of anterior deirid (ADE) neurons being well-structured neural circuits in the head region [35]. Neurodegeneration was scored by monitoring the fluorescent phenotyping of dopaminergic neurons in the *C. elegans* UA57 lines. Rotenone-induced senile nematodes showed degenerated dopaminergic neurons, with severe CEP and ADE neuronal death and partial axon breakage. *daf-12* RNAi rotenone-induced nematodes had almost loss of cell bodies and complete axonal degeneration (Fig. 3A). Loss of dopaminergic neurons and axonal breakage were significantly remedied by the VitD3 treatment (Fig. 3B), suggesting VitD-VDR signal exerting the protective effects on dopaminergic neurons in the *C. elegans* PD model.

**Fig. 4:**
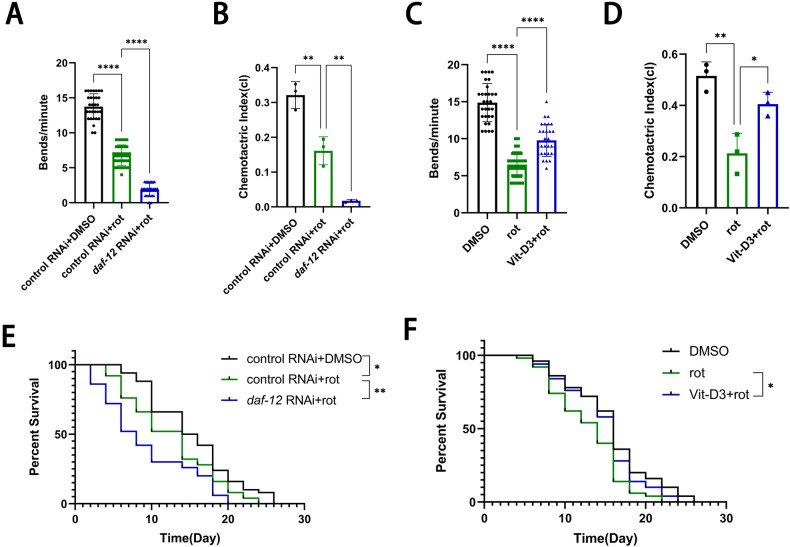
Neuron-specific knockdown *of daf-12* (VDR ortholog) aggravates motor deficits and shortens lifespan in the PD *C. elegans* model. **A** Body bends of neuronal RNAi-sensitive *C. elegans* Line TU3401 nematodes treated with *daf-12* knockdown and rotenone exposure for 8 days. *n* = 30 nematodes. **B** Chemotactic index of TU3401 strain after *daf-12* RNAi and control RNAi. *n* = 3 replicates (around 100 nematodes/each). **C** Body bends of Line TU3401 nematodes treated with VitD3 and rotenone exposure for 8 days. *n* = 30 nematodes. **D** Chemotactic index of TU3401 strain after VitD3 treatment. *n* = 3 replicates (around 100 nematodes/each). **E**, **F** Survival of TU3401 strain treated with *daf-12* knockdown (**E**) and Vitamin D3 (**F**) in rotenone exposure. *n* = 50 nematodes. Data presented as the mean ± SEM. *P < 0.05, **P < 0.01, ***P < 0.001 and ****P < 0.0001.

To further investigate the VDR effects on α-syn in *C. elegans*, we picked up the Strain NL5901 of α-syn stable expression with GFP florescence. *daf-12* RNAi enhanced α-syn expression, whilst VitD3 treatment reduced the α-syn levels (Fig. 3C, D). PD patients show clinical symptoms of movement disorders, including bradykinesia, static tremor, and myotonia. In *C. elegans*, dopamine controls motor coordination and food hunting (chemotaxis to the environmental ethanol and benzaldehyde). Disruption of dopamine signaling results in ethological changes; thus, motor and chemotaxis were used to evaluate the PD-like impairment of dopaminergic system in *C. elegans*. Compared with the wild-type Strain N2, the Strain NL5901 presented the low-frequency of body bends and poor chemotaxis, *daf-12* RNAi aggravated behavioral disorders (Fig. 3E, F), but VitD3 treatment improved motor coordination (Fig. 3G, H). In addition, another *C. elegans* Strain TU3401 was used to identify what effects of *daf-12*-specific knockdown in neurons. Frequency of body bends, chemotaxis, and lifespan were dramatically reduced after the rotenone treatment, similar to the α-syn-overexpressed strain. Neuron-specific *daf-12* knockdown further worsened the behavioral deficits and lifespan (Fig. 4A, B, E), whereas VitD3 treatment recovered the rotenone-induced movement disorders and extended the nematode lifespan (Fig. 4C, D, F). All these data indicate the positive roles of VitD-VDR in dopaminergic neuron, α-syn, and motor disorders in the PD *C. elegans* model.

### VitD-VDR enhances oxidative stress resistance via transcriptional regulation of downstream *gst* and *daf-16*

**Fig. 5:**
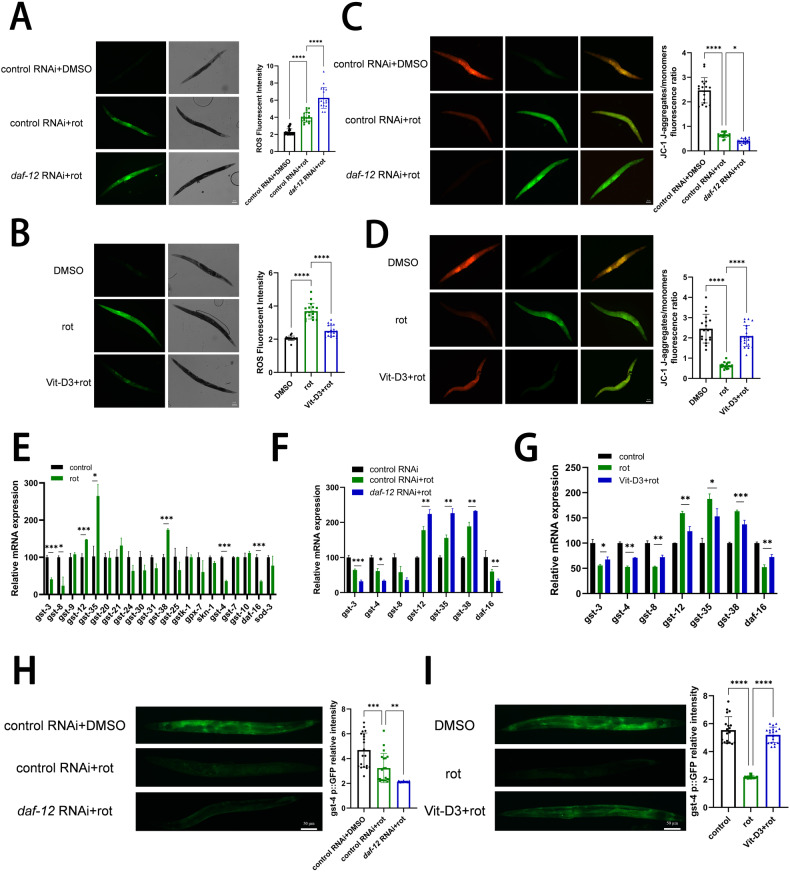
VDR alleviates mitochondrial oxidative stress through upregulation of *gst-4* and *daf-16.* **A**, **B** Representative images of ROS production in *C. elegans* Line TU3401 treated with *daf-12* knockdown (**A**) and VitD3 (**B**) with rotenone exposure of eight days, which was visualized with DCFDA fluorescence. **C**, **D** Representative images of JC-1 and quantity aggregates/monomers fluorescence intensity analysis. Scale bar, 50 μm. *n* = 20 nematodes. **E** Relative mRNA levels of genes relevant to antioxidative stress in TU3401 Line treated without or with rotenone. **F**, **G** Relative mRNA levels of genes in TU3401 Line treated with *daf-12* knockdown (**F**) and VitD3 (**G**) with rotenone exposure of 8 days. Each RNA sample was isolated from ~300 nematodes, *n* = 3 replicates. **H**, **I** Representative images of *gst-4P::GFP* expression in CL2166 and quantification of *gst-4P::GFP* relative fluorescence intensity analysis. Scale bar, 50 μm. *n* = 20 nematodes. Data presented as the mean ± SEM. *P < 0.05, **P < 0.01, ***P < 0.001, and ****P < 0.0001.

Beyond VDR protecting from oxidative stress in primary neurons, we found VDR also playing the same effects in *C. elegans*. Neuron-specific *daf-12* knockdown exacerbated rotenone-induced ROS and the MMP loss (Fig. 5A, C), but recovered by the VitD3 treatment (Fig. 5B, D). To further identify particularly VitD-VDR downstream events, various oxidative stress-related genes, such as *gst* (glutathione S-transferase), *gstk-1* (GST activity), *skn-1* (SKiNhead-1), *daf-16* (forkhead box transcription factor class O) and *sod-3* (superoxide dismutase), whose transcript levels were tested. Herein, rotenone significantly down-regulated the transcript levels of *gst-3*, *gst-4*, *gst-8,* and *daf-16*, and up-regulated the transcript levels of *gst-12*, *gst-35* and *gst-38* (Fig. 5E). Notably, *daf-12* RNAi further affected rotenone-induced transcriptional changes of genes related to antioxidation (Fig. 5F), and VitD3 treatment exerted the alleviating effects (Fig. 5G). Gene *gst-4* is widely accepted as an antioxidative marker in *C. elegans*. Stable *gst-4* expression in the Stain CL2166 was further reduced after rotenone treatment and *daf-12* RNAi, and recovered after VitD3 treatment (Fig. 5H, I). These *C. elegans* data indicate VitD-VDR pathway protects against mitochondrial dysfunction and oxidative stress, and *daf-12* expression in neurons plays an antioxidative role depending on the downstream *gst* and *daf-16* signals.

### DUB3 mediates VDR deubiquitination and plays the protective roles in neuron and microglia

**Fig. 6:**
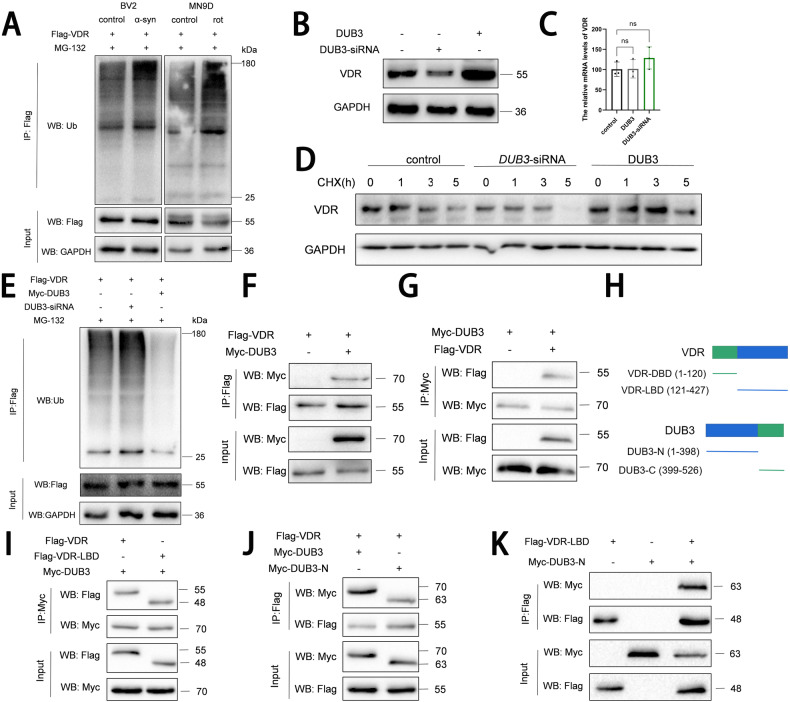
DUB3 mediates VDR deubiquitination regulation. **A** Western blotting analysis of ubiquitination level of VDR in MN9D neuron and BV2 microglial cell models, cells were treated with MG132 for 10 hours before harvested. **B** Representative immunoblots for VDR protein levels treated with VDR overexpressing and VDR knockdown BV2 cell lines. **C** The relative transcript levels of VDR in BV2 cell lines were detected by qRT-PCR. **D** BV2 cells were transfected with DUB3 plasmid and DUB3-siRNA followed by treatment with 50 μM cycloheximide (CHX) for the indicated times. **E** BV2 cells were transfected with Flag-VDR and Myc-DUB3 or DUB3-siRNA was then immunoprecipitated from denatured cell lysates. Cells were treated with 10 µM MG132 for 10 hours before collection. **F**, **G** Western blot analysis of co-immunoprecipitated proteins from BV2 cells transfected with Flag-VDR and Myc-DUB3. **H** Schematic diagram depicting a set of Flag-tagged VDR and Myc-tagged DUB3 constructs. **I** Full length or LBD domain of VDR and Myc-DUB3 were expressed in BV2 cells, immunoprecipitated, and analyzed by immunoblotting. **J** Full length or N-terminal of DUB3 and Flag-VDR were expressed in BV2 cells, immunoprecipitated, and analyzed by immunoblotting. **K** Flag-VDR-LBD and Myc-DUB3-N terminal were expressed in BV2 cells, immunoprecipitated, and analyzed by immunoblotting.

As the VDR protein levels were decreased in both rotenone-induced neuron and α-syn-induced microglia (Fig. S1), further the *daf-12* protein levels were increased after treatment of proteasome inhibitor MG132 in the *C. elegans* Line OH14589 (Fig. S6 A, B), we speculate whether VDR is under control of (de)ubiquitination. After ectopically expressed Flag-VDR, rotenone promoted VDR ubiquitination in dopaminergic neurons, and neuron-released α-syn also enhanced VDR ubiquitination in microglia (Fig. 6A, Fig. S8), suggesting VDR (de-)ubiquitination involved in the PD progression. In the deubiquitinase library, we screened out the deubiquitinating enzyme DUB3, whose overexpression up-regulated VDR proteins and prolonged the VDR protein half-life, but did not change the transcript levels of VDR. DUB3 knockdown achieved the contrary results (Fig. 6B–D, Fig. S8). Particularly, the decreased VDR ubiquitination was observed in the DUB3 expressed group, and intensified VDR ubiquitination after DUB3 knockdown (Fig. 6E, Fig. S8). Co-IP assay presented DUB3-VDR interaction (Fig. 6F, G, Fig. S8), validating DUB3-mediated VDR deubiquitination. As Fig. 6H shown, VDR contains N-terminal DNA-binding domain (DBD, 1–120 amino acids) and C-terminal ligand-binding domain (LBD, 121–427 amino acids) [36, 37]; DUB3 contains the N-terminal catalytic domain (1–398 amino acids) and C-terminal two HA binding region (399–526 amino acids) [38, 39]. We established the Flag-VDR-LBD and Myc-DUB3-N constructs, Co-IP assay demonstrated the DUB3-N terminal domain specific interacting with the VDR-LBD domain (Fig. 6I–K, Fig. S8). These suggest that DUB3 interacts with VDR and mediates deubiquitination to regulate the VDR protein levels.

**Fig. 7:**
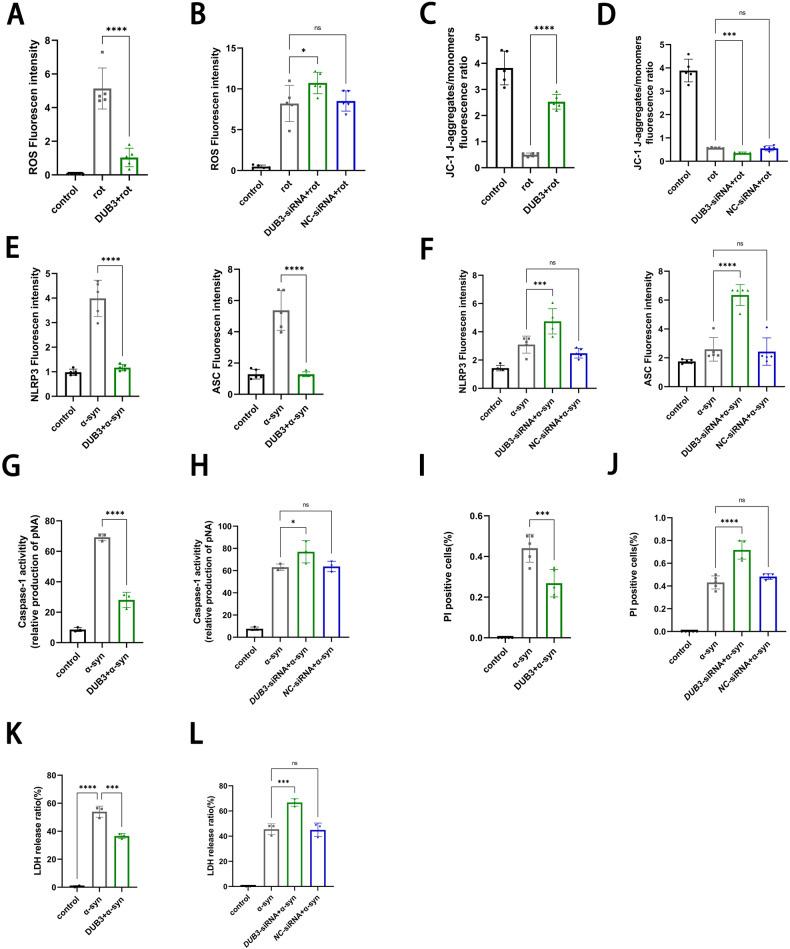
Protective effects of DUB3 on pathological models of primary neurons and microglia. **A**, **B** ROS fluorescent intensity of cells treated with or without rotenone in DUB3 overexpressing (**A**) and knockdown (**B**) primary neurons, ROS was labeled with a DCFH-DA probe. **C**, **D** JC-1 aggregates/monomers fluorescent ratio of primary neurons to assess the MMP. JC-1 experiments of DUB3 were parallelly performed with VDR experiments in Fig. 1. **E**, **F** NLRP3 and ASC Fluorescence in primary microglia treated with DUB3 overexpression (**E**) and DUB3 knockdown (**F**). **G**, **H** Caspase-1 activity of cells under the different treatments. **I**, **J** PI-positive cells of primary microglia subjected to different treatments. **K**, **L** LDH release of cells. DUB3 experiments in primary microglia were parallelly performed with VDR experiments in Fig. 2. Data present as the mean ± SEM; *n* ≥ 3 biologically independent replicates. *P < 0.05, **P < 0.01, ***P < 0.001, and ****P < 0.0001, ns, no significant difference.

Next, DUB3 function was investigated in both neurons and microglia. DUB3 overexpression in the primary neurons reversed the rotenone-induced ROS damage and MMP decrease, while DUB3 knockdown aggravated these effects (Fig. 7A–D). In the primary microglia, DUB3 manipulation regulated α-syn-induced NLRP3/ASC, caspase-1 activity, lactate dehydrogenase (LDH) release, and propidium iodide (PI) positive cells (Fig. 7E–L, Fig. S7), preventing inflammation and membrane permeability-induced microglial activation. The protective effects of DUB3 were consistent with that of VDR, suggesting that the DUB3-mediated VDR deubiquitination plays a role in neuroprotection in PD progression.

## Discussion

As the VitD-VDR pathway plays a widespread roles in dopaminergic neural circuits, VitD supplement is developed as PD adjuvant treatment [14, 16]. VDR plays protective roles in neuronal antioxidation and microglial anti-inflammation. The upstream DUB3-mediated VDR deubiquitination can maintain the high VDR protein levels and further inhibit mitochondrial oxidative stress and microglial activation, suggesting VDR is able to regulate the PD progression with multiple targets.

Mitochondrial dysfunction is one of the crucial factors in PD progression, and excessive oxidative stress contributes to neuronal death [2]. This study showed VDR overexpression protects neurons by reducing ROS and preserving mitochondrial function. In *C. elegans*, VDR was found to inhibit α-syn, which exert neuronal toxicity by damaging the mitochondrial complex, inducing endoplasmic reticulum stress, inhibiting proteasome/lysosome-mediated protein homeostasis and dopamine release [40–43]. Also, α-syn aggregates in neurons bind to NMDA receptor, facilitate membrane permeabilization and α-syn release, further applying to glial cells, promoting the toll-like receptors (TLR)-mediated glial activation and inflammation response involved in the PD pathological development [44–46]. Both primary neuron and nematode data strongly suggest VDR as a promising therapeutic target for alleviating oxidative stress and mitochondrial dysfunction in neurons.

VitD-VDR enhanced signaling protects from PD neuroinflammation. Our study supports that VDR overexpression inhibits α-syn-induced microglial activation, which was mediated by the NLRP3 inflammasome. VDR can down-regulate NF-κB activity by binding with the inhibitor of κB kinase (IKK), further blocking NF-κB-mediated NLRP3/caspase-1/GSDMD pyroptosis in embryonic fibroblasts and renal tubular cells [47, 48]. Another study found that VDR interferes with NLRP3 activation by disrupting deubiquitinase BRCC3-NLRP3 complex to promote NLRP3 degradation in bone marrow-derived macrophage [49]. These findings suggest that VDR provides multi-targeted protection for both neurons and microglia, offering a potential treatment for neuroinflammation in PD.

The ubiquitination-proteasome system is essential for the post-translational modification and VDR protein degradation. DUB regulates the deubiquitinating pathway, whose mechanism and function have been recently spotlighted in PD field [50, 51]. In this study, we identify a novel VDR deubiquitinase DUB3 and its molecular mechanism. The roles of deubiquitination in regulating VDR signal transduction as well as the downstream events of mitochondrial oxidative stress and neuroinflammation are supported. VDR consists of N-terminal DNA-binding domain (DBD), C-terminal ligand-binding domain (LBD), and a hinge region between the two domains. The DBD domain directs specific DNA-binding sites responsible for the VDR transcriptional modification, while the LBD domain and its adjacent hinge region interact with RXR to form dimers [36, 37]. DUB3 (also termed USP17) contains two functional domains: the N-terminal catalytic domain and two hyaluronan binding motifs in the C-terminal region. Catalytic triad carries conserved residues of Cys89, His334, and Asp350, responsible for deubiquitinating activity [52]. Our study indicates that DUB3-N-terminal catalytic domain interacts with the C-terminal LBD domain of VDR to mediate VDR deubiquitination. This finding elucidates the regulatory role of deubiquitination in VDR signaling and its downstream events, such as mitochondrial oxidative stress and neuroinflammation.

VDR belongs to the nuclear hormone receptor family of transcription factors and binds with RXR to form a heterodimer, which specifically binds to the VitD response elements (VDRE) of target genes, performing transcriptional regulation of downstream genes [21, 53]. Multiple lines of research have found that VitD treatment can increase the expression of voltage-gated calcium channel [54], catechol-o-methyltransferase [12], glial-derived neurotrophic factor [55], and tyrosine hydroxylase [11], where genes are essential for the protection of dopaminergic neuron. As VDR playing a role in oxidative resistance and preventing mitochondrial dysfunction, our nematode study identifies the VDR downstream candidates related to antioxidation. *Gst* is a large family involved in detoxification and antioxidation; herein, *gst-4* (human ortholog of hematopoietic prostaglandin D synthase (HPGDS)) is widely accepted as an antioxidative marker and plays a neuroprotective role in *C. elegans* of PD [56, 57]. *Daf-16* (human ortholog of forkhead box transcription factor class O) is newly found to involve in α-syn aggregation in nematode [34, 58–60]. Further studies need to clarify whether these *GST* genes as direct transcriptional targets of VDR, providing an understanding of VDR direct downstream regulation involved in oxidative stress resistance in the PD progression.

The present study reveals VitD-VDR able to alleviate neuronal oxidative stress, prevent loss of dopaminergic neuron, inhibit microglia activation, and improve motor function. Further, we identify the upstream signal DUB3, which stabilized high levels of VDR by deubiquitination. This rescue of neurodegeneration in the pathological model highlights the importance of DUB3 in VDR regulation. The discovery of DUB3 adds understanding of VDR deubiquitination system and improve the mechanism study of VDR functional upregulation. Multi-targeted regulatory effects of VDR, including antioxidation in dopaminergic neuron and anti-microglial activation, provide a potential target in PD prevention and treatment.

## Materials and methods

### Cell culture

#### Extraction and culture of primary microglia cells

SD rats were treated with ethanol, and the cerebral cortex was isolated. The cortex was digested with trypsin solution, and tissue fragments were dissociated through pipetting digestion was terminated by adding Dulbecco’s modified Eagle’s medium (DMEM) (Bosterbio, Wuhan, China) culture solution containing 10% fetal bovine serum (FBS) (Gibco, Grand Island, USA). Cells were centrifuged and resuspended in a medium, and then cultured at 37 °C with 5% CO_2_. After 48 hours, the culture medium was replaced regularly. When the primary cells reached 90% confluency, they were digested and transferred to a new culture flask. The nonadherent cells were seeded into another flask, while the adherent cells (microglia) were used for further experiments after 48 hours of culture.

#### Extraction and culture of primary neuron cells

C57 mice at 16 to 18 days gestation were anesthetized with 10% chloral hydrate. The fetal rat cerebral cortex was isolated and digested with trypsin solution. Tissue fragments were dissociated and mixed with trypsin solution, which was terminated by adding DMEM with 10% FBS. After filtration, cells were inoculated on well plates coated with polylysine and cultured in B27 supplemented neural base medium (Gibco, Grand Island, USA). After 6 hours, the medium was changed to Neurobasal medium supplemented with B27 (Solarbio, Beijing, China), glutamine, and Penicillin–Streptomycin (PS) (Beyotime, Shanghai, China). The medium was replaced every three days until cells (~9–10 days) ready for further experiments.

#### Culture of cell lines

MN9D mouse dopaminergic neuron cells were cultured in DMEM, supplemented with 10% fetal bovine serum (FBS) and PS (100 U/ml). BV2 mouse microglia cells were maintained in high-glucose minimal essential medium (MEM) supplemented with 10% FBS and PS (100 U/ml).

### Cell transfection

Cells were cultured until 50–70% density for transfection. After rinsing with PBS, serum residue was removed, and 900 μL of single culture medium was added. For transfection, 2 μg of siRNA or overexpressed plasmid, along with 2 μL of Lipofectamine 2000 reagent (Invitrogen, Carlsbad, USA), were mixed in 100 μL of Opti-MEM medium. After incubation and mixing with 1 mL of serum-free medium for 6 hours, the serum-free medium was replaced with 10% serum medium. Phenotype was confirmed 24 hours (plasmid overexpression) or 48 hours (siRNA transfection) later. siRNAs were designed and purchased by GenePhrama (Shanghai, China). siRNA sequences are shown in Table S2.

### Detection of reactive oxygen species (ROS) and mitochondrial membrane potential (MMP)

Reactive oxygen species (ROS) were detected according to DCFH-DA fluorescent probe instructions (Beyotime, Shanghai, China). Mitochondrial membrane potential was detected according to the instructions for JC-1 (Beyotime, Shanghai, China).

### Immunofluorescence

Cells were cultured in a confocal dish until 50%-60% confluency. Cells were washed with PBS and fixed with 4% paraformaldehyde. After rinsing with 1% Triton solution, cells were permeabilized for 10 minutes. Then, cells were blocked with 4% BSA for one hour. The corresponding NLRP3 antibody (Proteintech, Wuhan, China) and ASC antibody (Affinity, Cincinnati, USA) were added and incubated overnight at 4 °C. After incubation, appropriate fluorescent secondary antibodies were applied and incubated. DAPI staining solution (Beyotime, Shanghai, China) was added for 10 minutes. Anti-fluorescence quencher was added and cells were examined under a Leica DMi8 inverted fluorescence microscope (Leica, Wetzlar, Germany).

### Propidium iodide (PI) staining

PI (Solarbio, Beijing, China) was diluted to the concentration of 10–50 µM (6.7–33.4 µg/mL). The incubation process was carried out according to the manufacturer’s instructions.

### Lactate dehydrogenase (LDH) release and caspase-1 activity assay

Cells were inoculated into the 96-well cell culture plate. Measurement of LDH release was conducted using the LDH release quantification cytotoxicity Assay Kit (Beyotime, Shanghai, China) as per the manufacturer’s instructions. To measure the caspase-1 activity, the provided instructions from Beyotime (Shanghai, China) were followed. PNA in the kit was diluted to create a range of standard solutions with different concentrations. A determination system was prepared by mixing the diluted PNA with the lysed cell suspension in a 1:9 ratio. A standard curve was generated using the prepared standard solutions. Following the kit instructions, a buffer system was prepared, and the absorbance at 405 nm was measured using an enzyme reader.

### Western blotting

Total protein was extracted by Cell lysis buffer for Western and IP (Beyotime, Shanghai, China) and quantified by the BCA methods. Protein bands were separated by SDS‐PAGE and then transferred to a polyvinylidene difluoride (PVDF) membrane. Finally, the bands were visualized with an enhanced chemiluminescence (ECL) Kit (Yeasen, Shanghai, China) using an Image Quant LAS 4000 mini (GE). Information on antibodies is shown in Table S1.

### Co-immunoprecipitation (Co-IP) assay

Cells were lysed in IP buffer (1 mM phenylmethylsulfonyl fluoride). Cell lysates were incubated with the indicated antibody and protein G-A garose beads (Yeasen, Shanghai, China) at 4 °C overnight. Then, the beads were washed three times with 1 mL IP buffer containing at 4 °C. The precipitates were analyzed by standard western blotting.

### Ubiquitination assay

Cells were harvested in 1% serum medium containing 5 µM MG132 (Selleck, Houston, USA) and treated for 10 hours. Cells were then lysed using IP buffer (50 mM Tris-HCl, pH 7.4, 150 mM NaCl, 1% Triton X-100, 1% sodium deoxycholate, and 1% protease inhibitor cocktails) on ice. Cell lysate was centrifuged, and the supernatant was incubated with primary antibodies and protein A/G agarose beads (Yeasen, Shanghai, China), rotating at 4 °C overnight. On the following day, the pellet was washed at least six times with 1× IP buffer on ice to remove non-specific binding. The washed pellet was then subjected to western blotting analysis to detect the protein of interest.

### Protein half-life analysis

Cells were treated with 50 μM CHX (Selleck, Houston, USA) at the indicated time points 24 hours after transfection. Cell lysates were analyzed using standard western blotting.

### Quantitative RT‐PCR

Total cell RNA in each group was extracted with Trizol reagent (Yeasen, Shanghai, China), and the RNA concentration was measured using a spectrophotometer (Nano Vue). Subsequently, the total RNA was reverse‐transcribed into Revert Aid First Strand cDNA Synthesis Kit (Omega, Doraville, USA) according to the manufacturer’s instructions. β-actin was used as the housekeeping genes. The primer sequences used for RT‐qPCR are shown in Table S2.

### C. elegans strains

All *C. elegans* strains used in this study are shown in Table S3. *C. elegans* transgenic strains were obtained from *Caenorhabditis* Genetics Center (University of Minnesota, Minneapolis, MN, USA).

#### *C. elegans* RNAi and drug treatments

*E. coli* strain HT115 was grown in LB containing 100 µg/mL ampicillin, tetracycline, and 100 µg/mL isopropyl 1-thio-β-D-galactopyranoside. L1 larval nematodes were placed on *daf-12* RNAi *E. coli* (Univ, Shanghai, China) or vector control plates at 19°C, then adult nematodes were synchronized on the first day. L1 larval nematodes were placed on *daf-12* RNAi or vector control plates at 19°C, HT115 expressing empty vector L4440 was used as the control RNAi. RNAi efficacy was determined by assay of qRT-PCR. For experiments with rotenone or vitamin D3 treatments, nematodes were transferred on L1 to nematode growth medium (NGM) plates seeded with 1 mL of heat-killed OP50 bacteria and 6 µM of rotenone, 400 µM of vitamin D3 or vehicle (0.5% DMSO). Each plate is coated with 100 µM fluorodeoxyuridine (FUDR) (Solarbio, Beijing, China).

#### Detection of locomotor ability of nematodes

*C. elegans* Lines of TU3401, N2, and NL5901 [61] were selected to test the motor ability. All nematode populations were cultured at 20 °C and developed synchronously for 4 hours. Individuals were transferred to FUDR plate 64–72 hours after oviposition, nematodes were collected on adult day 8, cleaned twice with M9 buffer, then transferred to fresh NGM plates for 1 minute adaptation, and the number of body bending of nematodes within 1 min was recorded.

#### Lifespan assay

*C. elegans* were transferred to NGM plates or RNAi-seeded NGM containing 6 μM rotenone. The numbers of live and dead worms were counted and recorded every other day. And the lifespan was calculated until all worms died.

#### Determination of neurodegeneration

*C. elegans* Line of UA57 [62] was fixed by adding 10 μL 40 mM levamisole buffer on a 2% agarose mat. Images were obtained on a confocal microscope at ×20 magnification, and the acquired z-stack images were processed using Zeiss ZEN software. A stationary region was mapped around each CEP and ADE dopaminergic cell body. Prior to scoring, a threshold area (µM^2^) was defined to define cell bodies as degenerate, and all cell bodies were subsequently counted as present if their area exceeded this predetermined threshold. The nerve axons protruding forward from the CEP cell body are counted if they do not exhibit degenerative morphology, such as blistering or chipped.

#### Detection of chemotaxis ability

Agar plates were divided into four quadrants. 1 μL 0.25 M sodium azide was mixed in the same parts with ethanol (95%) as control, or odorant (0.1% benzaldehyde in 100% ethanol) as attractant. Either 2 μL of control or attractant solution was added to the center of two opposite quadrants with the same distance to the middle of the plate. Nematodes were washed and separated from larvae, as stated above, and a number of approximately 150 animals placed in the plates’ center. After 1 h, each quadrant was counted, and a chemotaxis index calculated ((number of attractant – number of control)/number total).

### Statistical analysis

All values were expressed as means ± the standard error of the mean (SEM). Statistical analyses were performed using One-way analysis of variance (ANOVA) and a post hoc test for multiple comparisons, conducted using SPSS v.22.0 (IBM, Armonk, NY, USA). *P* values < 0.05 are considered statistically significant. Each experiment was repeated with at least three independent biological replicates.

### Supplementary information

supplementary figures and tables

Figure S1. VDR expression in the PD cell model.

Figure S2. VDR alleviates rotenone-induced mitochondrial dysfunction in MN9D cells.

Figure S3. siRNA interference efficacy.

Figure S4. VDR inhibits α-syn-induced inflammatory response in microglia BV2 cell line.

Figure S5. VDR inhibits α-syn-induced microglial permeabilization in BV2 cell line.

Figure S6. daf-12 under ubiquitinated regulation in C. elegans.

Figure S7. DUB3 plays protective roles in primary neurons and microglia.

Figure S8. Full-length uncropped original western blots

## Data Availability

All datasets generated for this study are included in the article/supplementary material.
