# Supplementary material for: VDR and deubiquitination control neuronal oxidative stress and microglial inflammation in Parkinson’s disease
Source: Cell Death Discov. 2024 Mar 21;10:150. doi: 10.1038/s41420-024-01912-9 (PMC10957901; doi:10.1038/s41420-024-01912-9)
Supplement: Supplementary file 1 — supplementary figures and tables [file 41420_2024_1912_MOESM1_ESM.pdf]

Supplementary figures

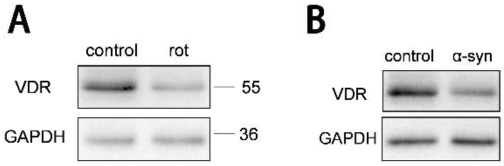

Figure S1. VDR expression in the PD cell model. (A) Representative immunoblots of VDR protein from primary neuronal cells treated without (control) or with rotenone (rot, 2 μM, 24 hour). **(B)** Representative immunoblots for VDR proteins from primary microglia treated with or without the medium from α-syn-overexpressed MN9D culture.

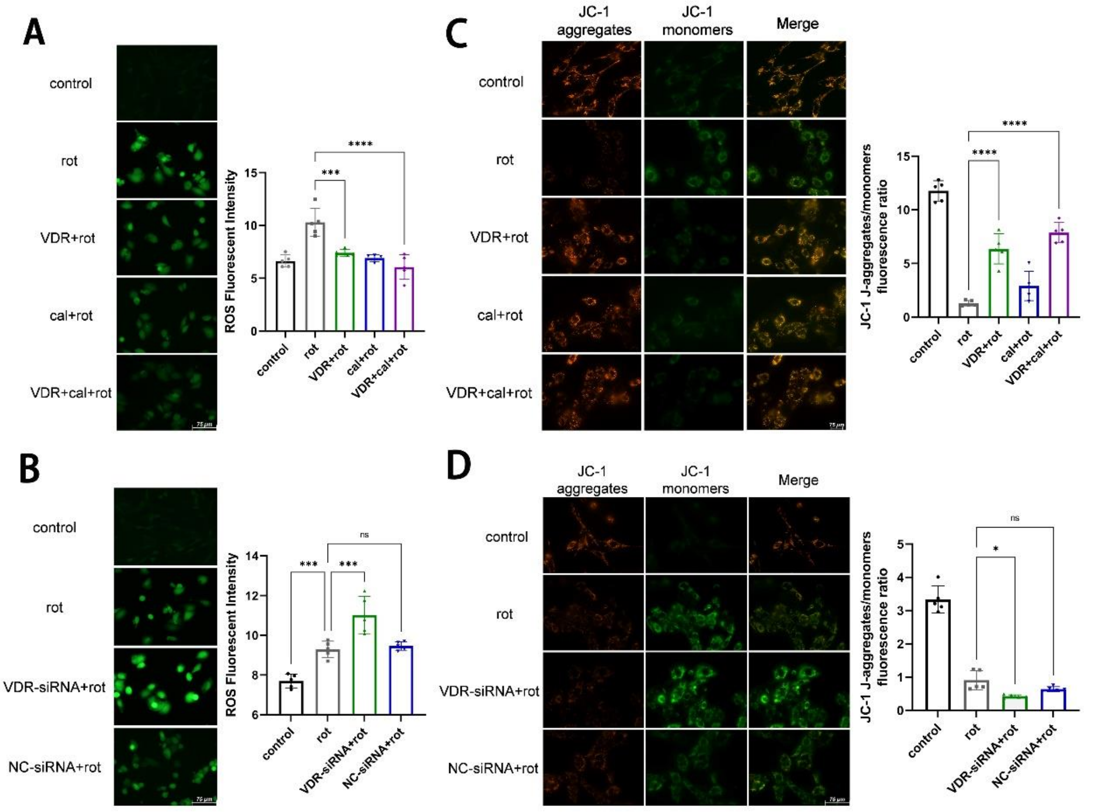

Figure S2. VDR alleviates rotenone-induced mitochondrial dysfunction in MN9D cells. (A, B) Fluorescence images of dopaminergic neuron cell line MN9D treated with rotenone, calcitriol in VDR overexpression (A) and VDR-siRNA knockdown (B), ROS was labeled with a DCFH-DA probe. The bar charts represent ROS fluorescence intensity analysis. Scale bar, 75 μm. **(C, D)** Fluorescence images of JC-1 (JC-1 aggregate, red; JC-1 monomer, green) stained MN9D cells to assess the MMP. Scale bar, 75 μm. Data present as the mean ± SEM; n=5 replicates. *P<0.05, ***P<0.001 and ****P<0.0001, ns, no significant difference.

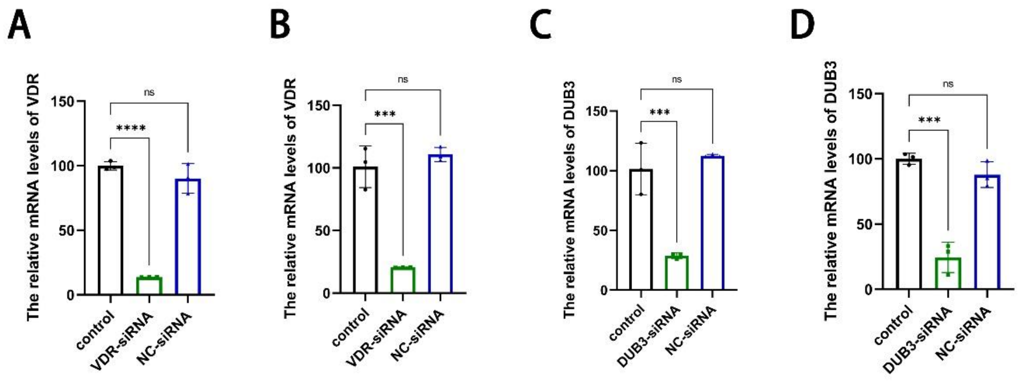

Figure S3. siRNA interference efficacy. qRT-PCR detected the knockdown efficacy of VDR-siRNA in primary neurons **(A)** and primary microglia **(B)**; the knockdown efficacy of DUB3-siRNA in primary neurons **(C)** and primary microglia **(D).** n=3 replicates. ***P<0.001 and ****P<0.0001, ns, no significant difference.

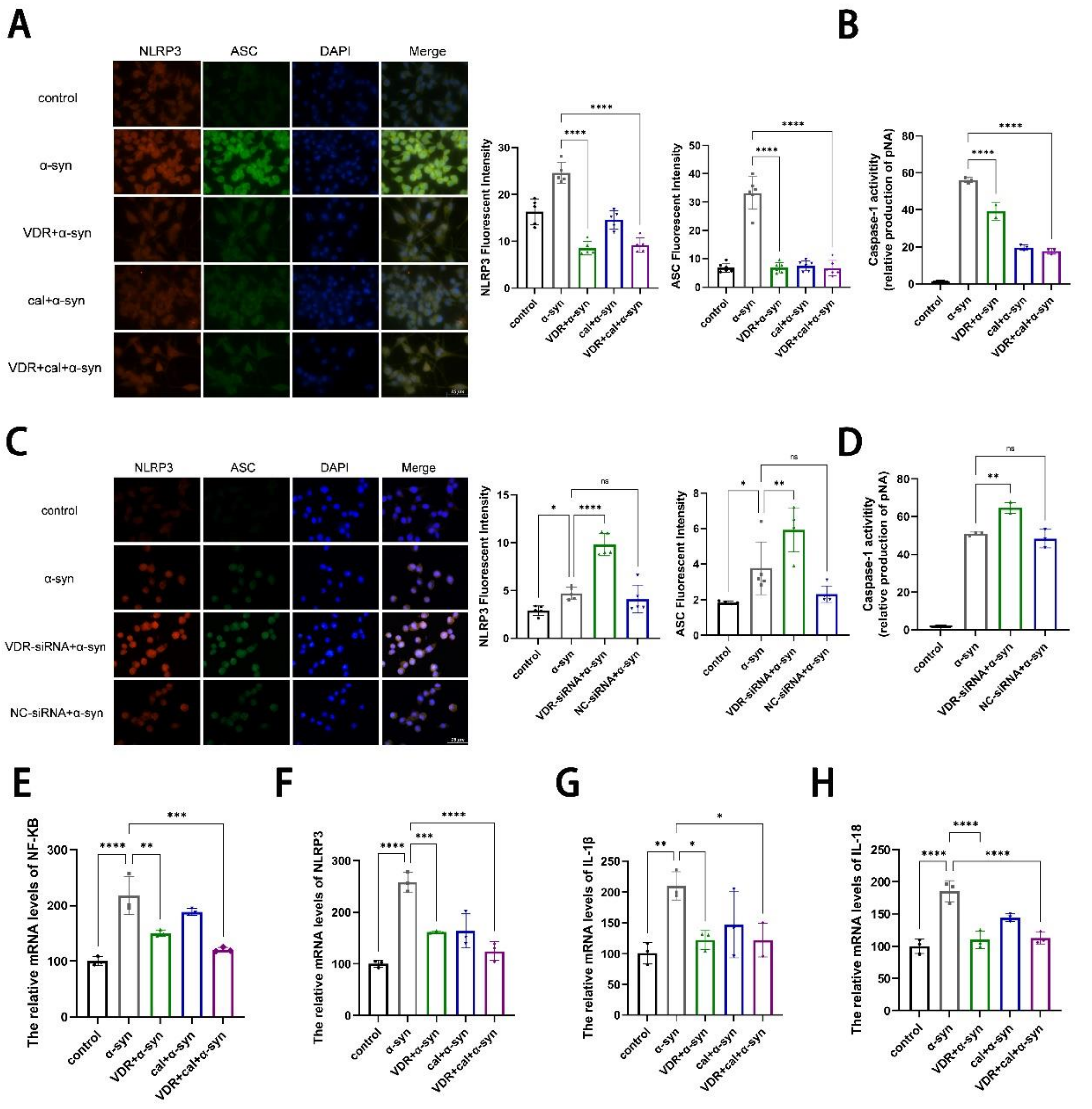

Figure S4. VDR inhibits α-syn induced inflammatory response in microglia BV2 cell line. (A, C) Fluorescent images showing microglia BV2 cell line treated with medium of α-syn-overexpressed MN9D followed by calcitriol (100 nM, 24 hours) treatment, VDR overexpression (A) and VDR-siRNA knockdown (C) in BV2 cell, labeled with DAPI (blue), NLRP3 (red) and ASC (green) as well as the overlay (yellow). The bar charts represent quantification of NLRP3 and ASC fluorescence intensity analysis. Scale bar, 75 μm. **(B, D)** Caspase-1 activity of BV2 cell line under the different treatments. **(E-H)** The relative transcript levels of NF-κB, NLRP3, IL-1β,

IL-18 detected by qRT-PCR. Data present as the mean ± SEM; n⩾3 biologically independent replicates. *P<0.05, **P<0.01, ***P<0.001, ****P<0.0001, ns, no significant difference.

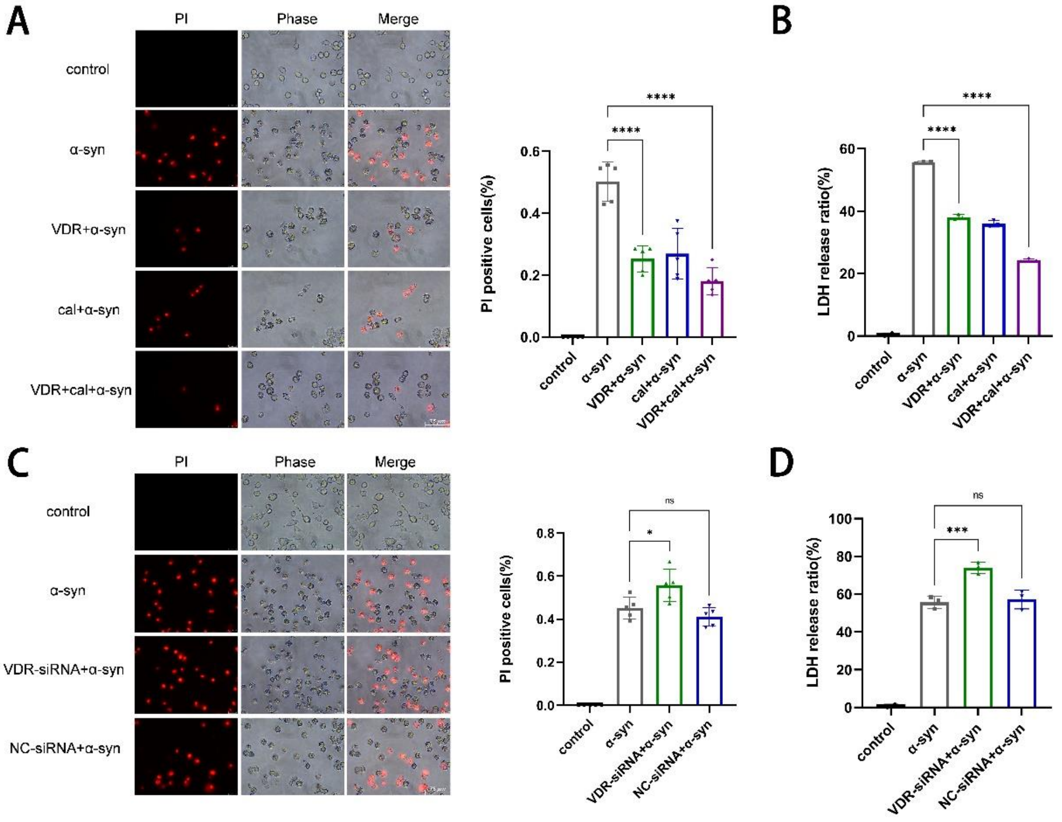

Figure S5. VDR inhibits α-syn induced microglial permeabilization in BV2 cell line. **(A, C)** Representative images of BV2 cell line subjected to different treatments. Cell membrane permeabilization was monitored by PI uptake (red fluorescence). Bar charts represent the percentage of PI positive cells. Scale bar, 75 μm. **(B, D)** LDH release detected by the kit to verify cell membrane integrity. Data present as the mean ± SEM; n⩾3 biologically independent replicates. *P<0.05, ****P<0.0001, ns, no significant difference.

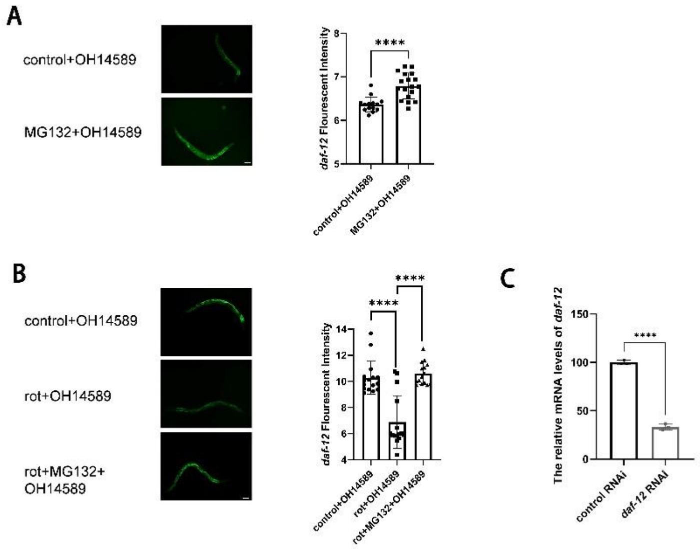

Figure S6. *daf-12* under ubiquitinated regulation in *C. elegans*. (A, B) The *C. elegans* Line OH14589 (expressing *daf-12::GFP::3×Flag* with endogenous *daf-12* promoter) was used to detect *daf-12* protein changes after the treatments of proteasome inhibitor MG132, or (and) rotenone. n = 15 nematodes. **(C)** qRT-PCR detected the knockdown efficacy of *daf-12* RNAi in NL2099 strain of *C. elegans*. n = 3 biologically independent replicates. Data present as the mean ± SEM, ****$P<0.0001$.

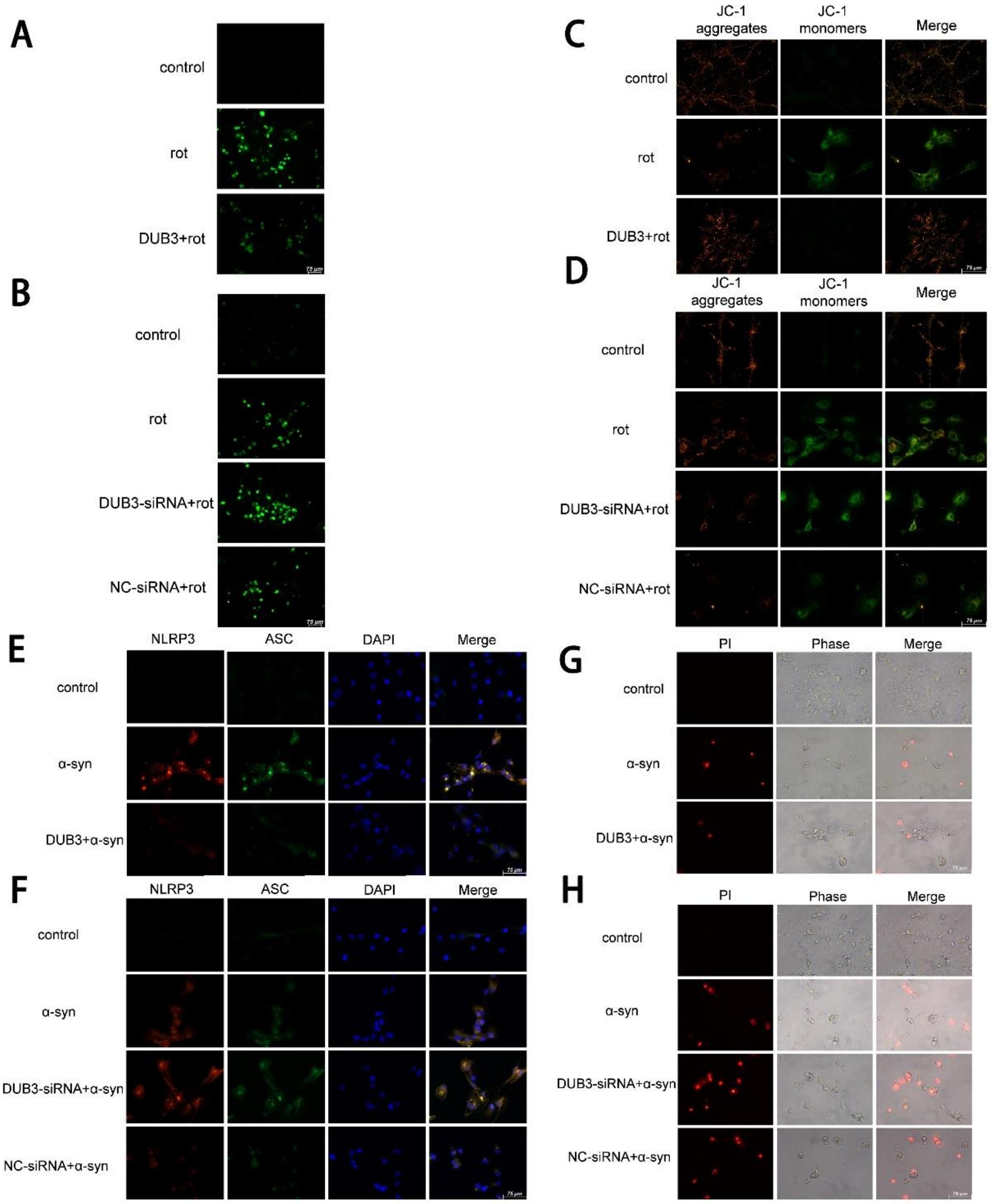

Figure S7. DUB3 plays protective roles in primary neurons and microglia. Fluorescent images corresponding to Figure 7. **(A, B)** Fluorescence images of primary neuronal cells treated with rotenone, calcitriol in DUB3 overexpression (A) and DUB3-siRNA knockdown (B), ROS was labeled with a DCFH-DA probe. Scale bar, 75 μm. **(C, D)** Fluorescence images of JC-1 (JC-1 aggregate, red; JC-1 monomer, green) stained primary neuronal cells to assess the MMP. Scale bar, 75 μm. **(E, F)** Fluorescent

images showing primary microglia treated with medium of α-syn-overexpressed MN9D cell, DUB3 overexpression (E) and DUB3-siRNA knockdown (F) in primary microglia labeled with DAPI (blue), NLRP3 (red) and ASC (green) as well as the overlay (yellow). Scale bar, 75 μm. **(G, H)** Representative images of primary microglia line subjected to different treatments. Cell membrane permeabilization was monitored by PI uptake (red fluorescence). Scale bar, 75 μm.

Figure S8. Full length uncropped original western blots

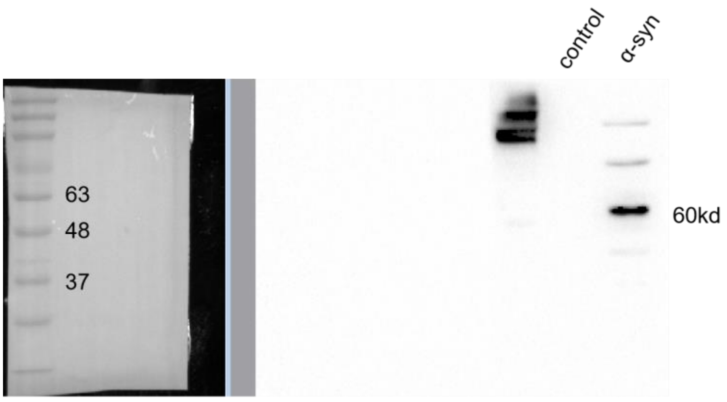

Corresponding to Fig 2A. WB: α-synuclein aggregates-60kd. GoldBand Plus 3-color Regular Range Protein Marker (8-180 kDa) was purchased from Yeasen (Shanghai, China). Synuclein alpha Ab was purchased from Affinity Biosciences (Cincinnati, USA).

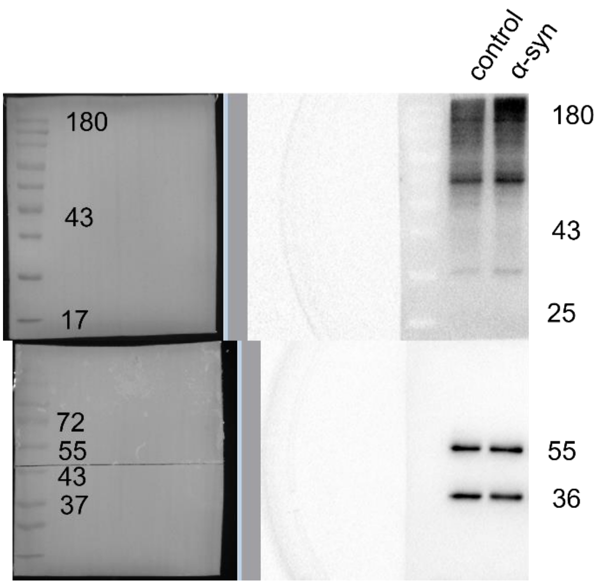

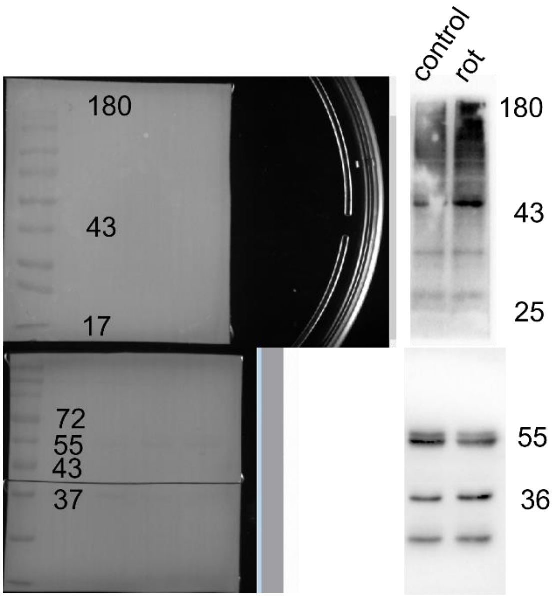

Corresponding to Fig 6A. WB: Ubiquitin, WB: VDR-55kd

GoldBand Plus 3-color Regular Range Protein Marker (8-180 kDa) was purchased from Yeasen (Shanghai, China). Purified anti-Ubiquitin Antibody was purchased from Biolegend (San Diego, USA). Vitamin D Receptor Antibody was purchased from Affinity Biosciences (Cincinnati, USA).

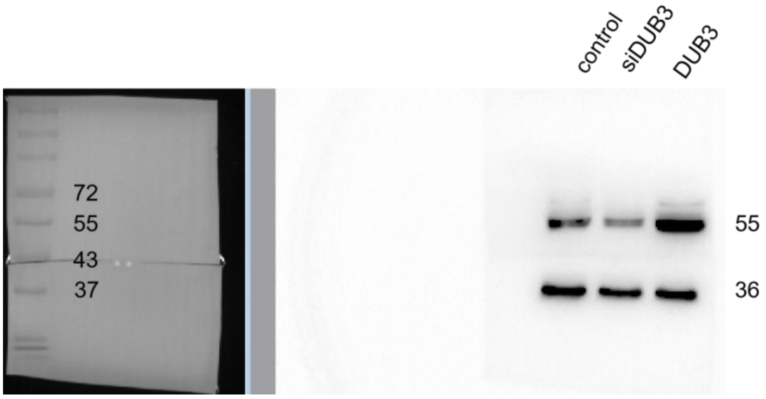

Corresponding to Fig 6B. WB: VDR-55kd

GoldBand Plus 3-color Regular Range Protein Marker (8-180 kDa) was purchased from Yeasen (Shanghai, China). Vitamin D Receptor Antibody was purchased from Affinity Biosciences (Cincinnati, USA).

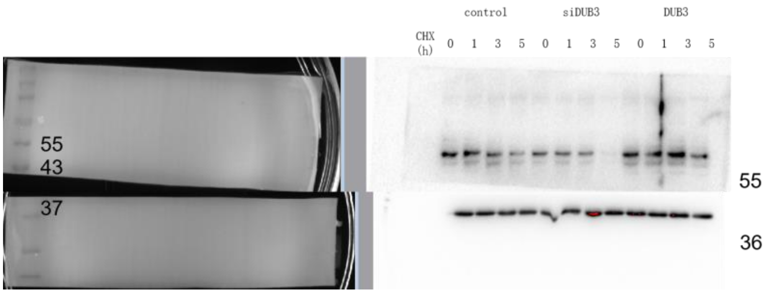

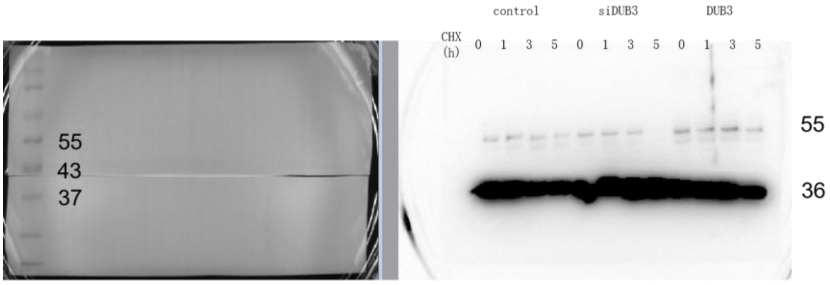

Corresponding to Fig 6D. WB: VDR-55kd

GoldBand Plus 3-color Regular Range Protein Marker (8-180 kDa) was purchased from Yeasen (Shanghai, China). Vitamin D Receptor Antibody was purchased from Affinity Biosciences (Cincinnati, USA).

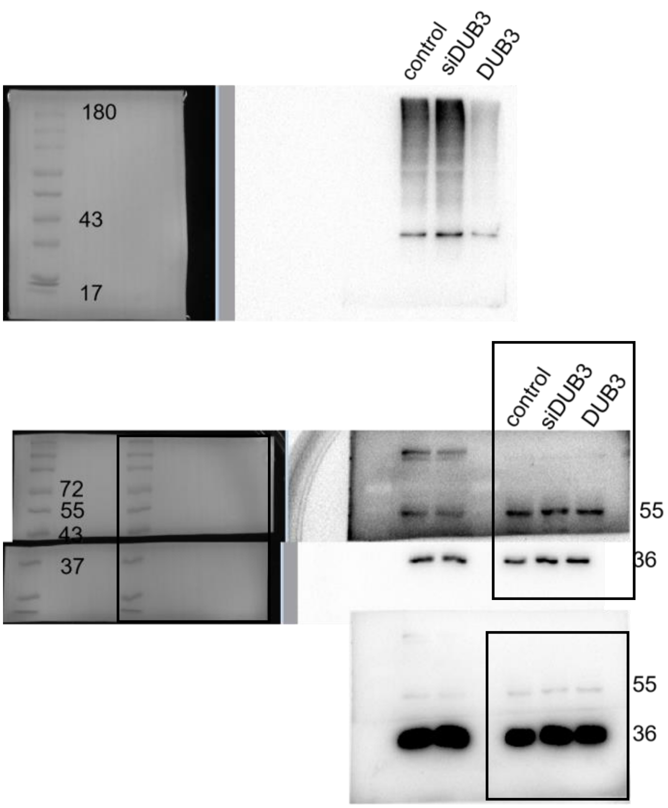

Corresponding to Fig 6E. WB: ubiquitin, VDR-55kd

GoldBand Plus 3-color Regular Range Protein Marker (8-180 kDa) was purchased from Yeasen (Shanghai, China). Purified anti-Ubiquitin Antibody was purchased from Biolegend (San Diego, USA). Vitamin D Receptor Antibody was purchased from Affinity Biosciences (Cincinnati, USA).

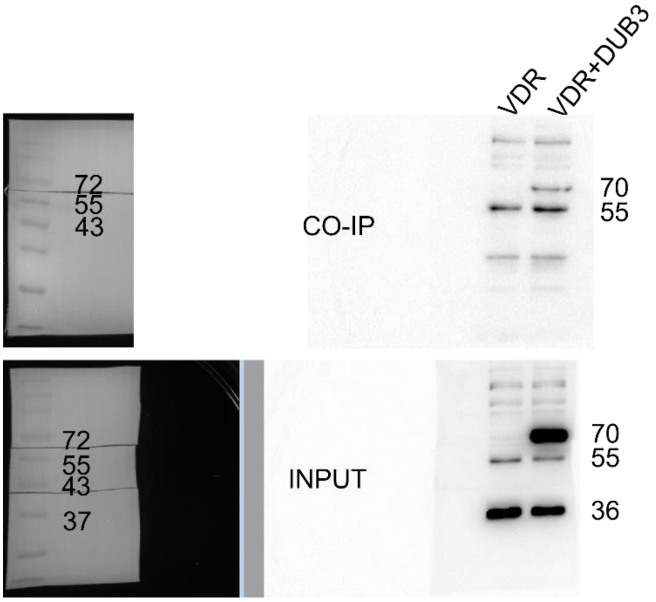

Corresponding to Fig 6F. WB: Myc-DUB3 70kd, Flag-VDR 55kd

GoldBand Plus 3-color Regular Range Protein Marker (8-180 kDa) was purchased from Yeasen (Shanghai, China). ColorMixed Protein Marker (11-180 KD) was purchased from Solarbio (Beijing, China). MYC Tag Polyclonal antibody was purchased from Proteintech (Chicago, USA). Flag Tag Antibody was purchased from Affinity Biosciences (Cincinnati, USA).

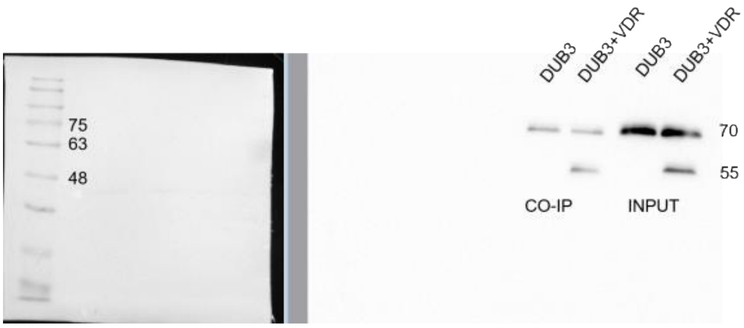

Corresponding to Fig 6G. WB: Myc-DUB3 70kd, Flag-VDR 55kd

GoldBand Plus 3-color Regular Range Protein Marker (8-180 kDa) was purchased from Yeasen (Shanghai, China). ColorMixed Protein Marker (11-180 KD) was purchased from Solarbio (Beijing, China). MYC Tag Polyclonal antibody was purchased from Proteintech (Chicago, USA). Flag Tag Antibody was purchased from Affinity Biosciences (Cincinnati, USA).

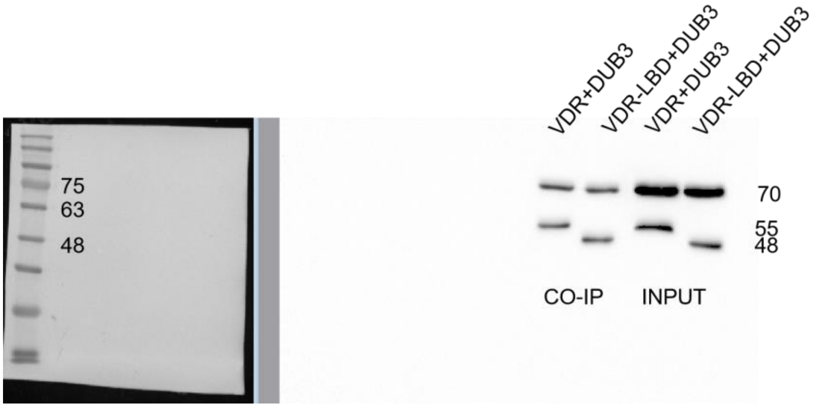

Corresponding to Fig 6I. WB: Myc-70kd, Flag-VDR 55kd, VDR-LBD 48kd

ColorMixed Protein Marker (11-180 KD) was purchased from Solarbio (Beijing, China). MYC Tag Polyclonal antibody was purchased from Proteintech (Chicago, USA). Flag Tag Antibody was purchased from Affinity Biosciences (Cincinnati, USA).

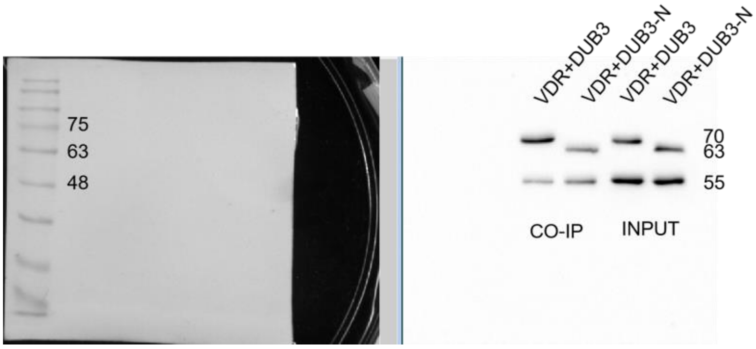

Corresponding to Fig 6J. WB: Myc-DUB3 70kd, DUB3-N 63kd, Flag-VDR 55kd

ColorMixed Protein Marker (11-180 KD) was purchased from Solarbio (Beijing, China). MYC Tag Polyclonal antibody was purchased from Proteintech (Chicago, USA). Flag Tag Antibody was purchased from Affinity Biosciences (Cincinnati, USA).

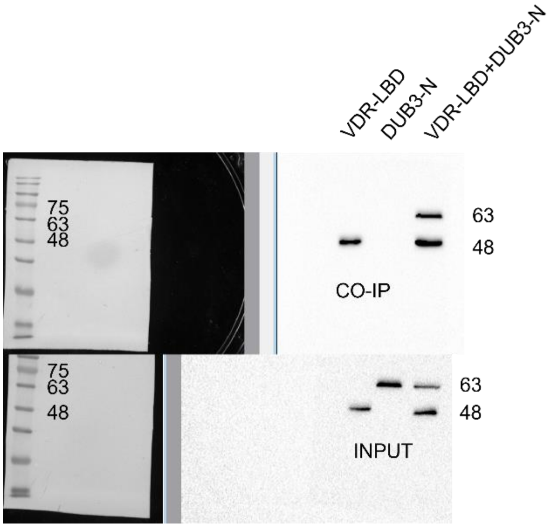

Corresponding to Fig 6K. WB: Myc-DUB3-N 63kd, Flag-VDR-LBD 48kd

ColorMixed Protein Marker (11-180 KD) was purchased from Solarbio (Beijing, China). MYC Tag Polyclonal antibody was purchased from Proteintech (Chicago, USA). Flag Tag Antibody was purchased from Affinity Biosciences (Cincinnati, USA).

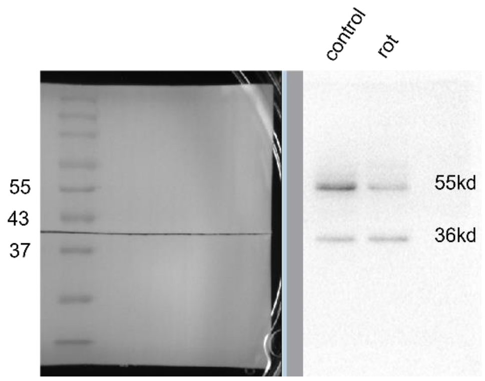

Corresponding to Fig S1A. WB: VDR-55kd

GoldBand Plus 3-color Regular Range Protein Marker (8-180 kDa) was purchased from Yeasen (Shanghai, China). ColorMixed Protein Marker (11-180 KD) was purchased from Solarbio (Beijing, China). Vitamin D Receptor Antibody was purchased from Affinity Biosciences (Cincinnati, USA).

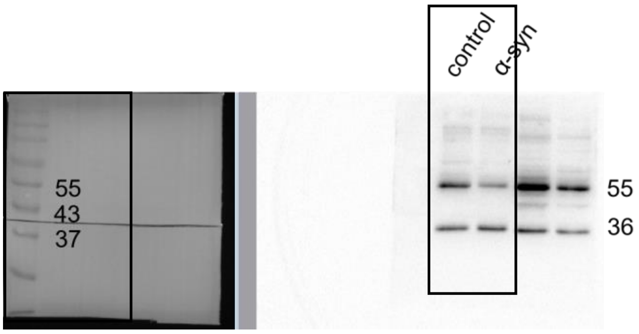

Corresponding to Fig S1B. WB: VDR-55kd

GoldBand Plus 3-color Regular Range Protein Marker (8-180 kDa) was purchased from Yeasen (Shanghai, China). ColorMixed Protein Marker (11-180 KD) was purchased from Solarbio (Beijing, China). Vitamin D Receptor Antibody was purchased from Affinity Biosciences (Cincinnati, USA).

Table S1. Antibodies

Antibody	Manufacturer
α-synuclein polyclonal antibody	Affinity (Cincinnati, USA)
ASC/TMS1 polyclonal antibody	Proteintech (Chicago, USA)
NLRP3 polyclonal antibody	Affinity (Cincinnati, USA)
VDR polyclonal antibody	Affinity (Cincinnati, USA)
Myc-Tag antibody	Proteintech (Chicago, USA)
Flag-Tag antibody	Affinity (Cincinnati, USA)
GAPDH antibody	Bosterbio (Wuhan, China)
Ubiquitin antibody	Biolegend (San Diego, USA)

Table S2. siRNA sequences and qRT-PCR primer sequences

	F-Primer	R-Primer
Actin	TCAACACCCCAGCCATGTAC	GCACGATTTCCCTCTCAGCT
VDR	AGTCAAGTGCCATTGAGG	ATGCAGATGGCCATGAGCAG
DUB3	GGACCTTTGCCTTATGCCCT	CTGTTTGAGGTCGGTCTGCT
Daf-12	AGGCGTTTCGTCAAAGTTGC	CTCCTCGTCGAAGAAACCGA
VDR-siRNA	GUCAGUUACAGCAUCCAAATT	UUUGGAUGCUGUAACUGACTT
DUB3-siRNA	CACCUCACAUACCUUCGAUTT	AUCGAAGGUAUGUGAGGUGTT

Table S3. *C. elegans* transgenic lines

Name	Transgene	Phenotype
N2	Wild-type	Wild type
UA57	dat-1p::GFP + dat-1p::CAT-2	GFP expression in CEP, ADE and PDE neurons.
NL5901	unc-54p::alphasynuclein::YFP + unc-119(+)	YFP expression in the muscles.
TU3401	N/A	Hypersensitive neuronal RNAi by feeding
NL2099	*snb-1*::Aβ1-42::3'UTR + *mtl-2*::GFP + *ida-1*p::GFP	Increased sensitivity to RNAi when compared to WT animals.
CL2166	*(pAF15)gst-4p::GFP::NLS*	Oxidative stress-inducible *gst-4* GFP.
OH14589	*daf-12::GFP::3×Flag*	*daf-12* overexpression with endogenous *daf-12* promoter

C. elegans transgenic strains were obtained from *Caenorhabditis* Genetics Center (University of Minnesota, Minneapolis, MN, USA).
