# Supplementary figures and images for: VDR and deubiquitination control neuronal oxidative stress and microglial inflammation in Parkinson’s disease
Source: Cell Death Discov. 2024 Mar 21;10:150. doi: 10.1038/s41420-024-01912-9 (PMC10957901; doi:10.1038/s41420-024-01912-9)

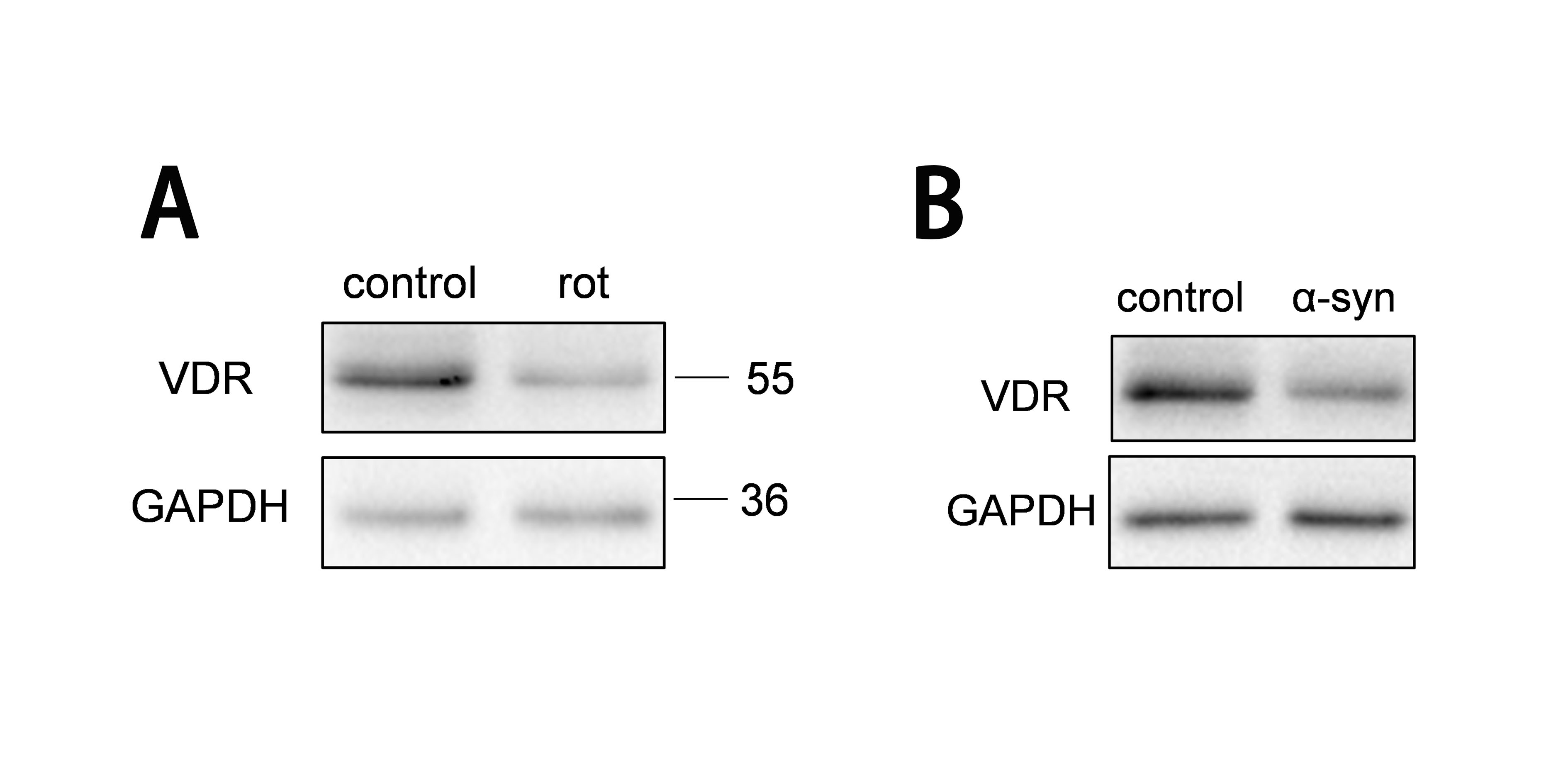

Supplement: Supplementary file 2 — Figure S1. VDR expression in the PD cell model. [file 41420_2024_1912_MOESM2_ESM.jpg]

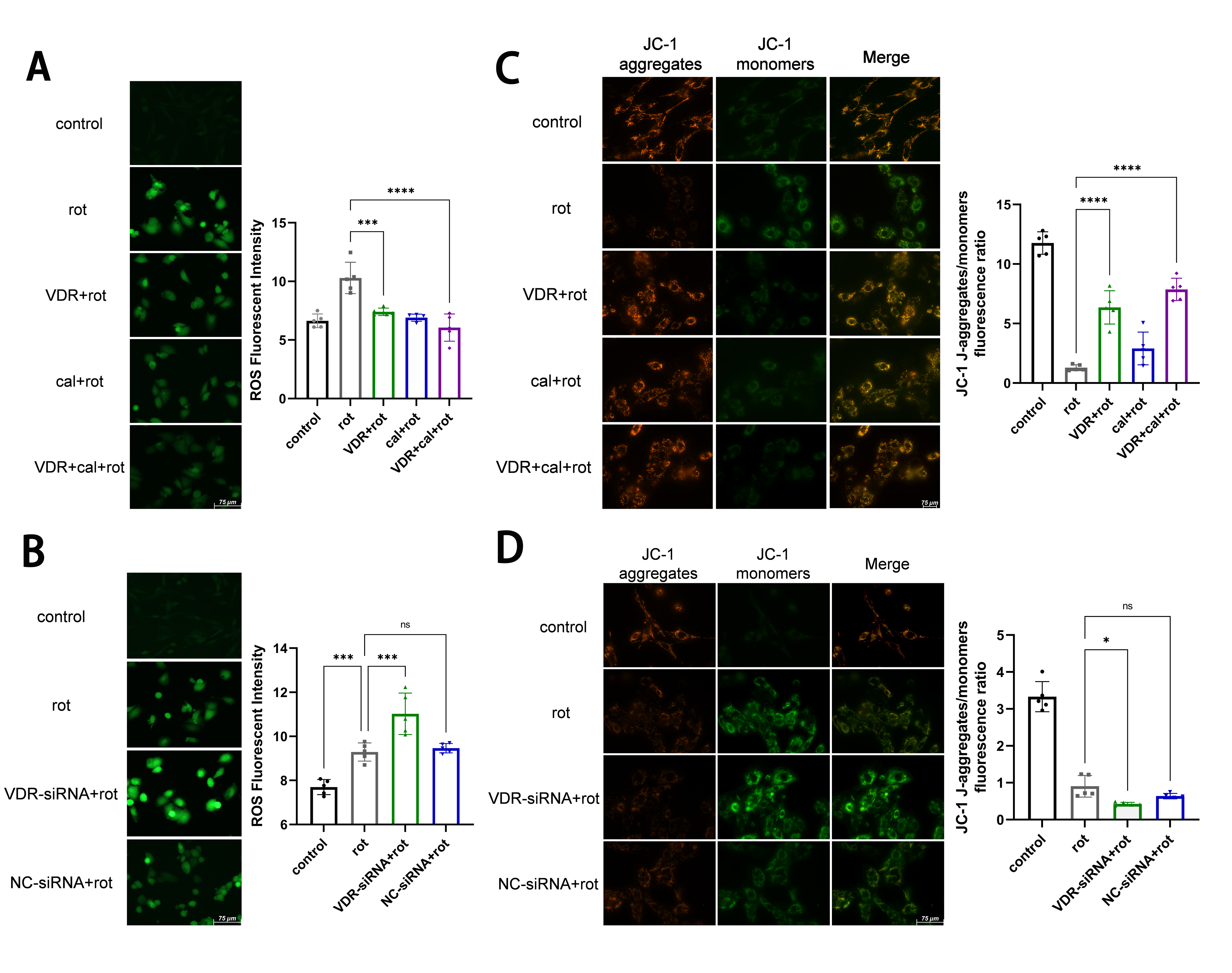

Supplement: Supplementary file 3 — Figure S2. VDR alleviates rotenone-induced mitochondrial dysfunction in MN9D cells. [file 41420_2024_1912_MOESM3_ESM.jpg]

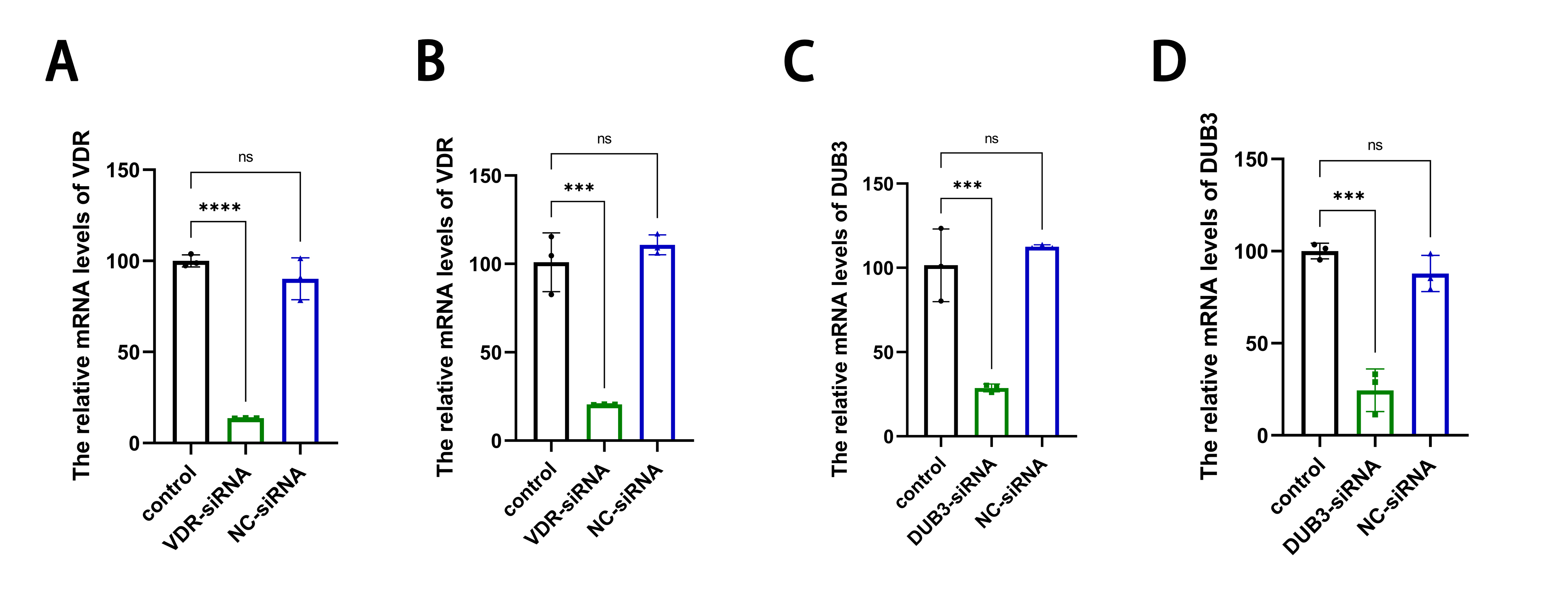

Supplement: Supplementary file 4 — Figure S3. siRNA interference efficacy. [file 41420_2024_1912_MOESM4_ESM.jpg]

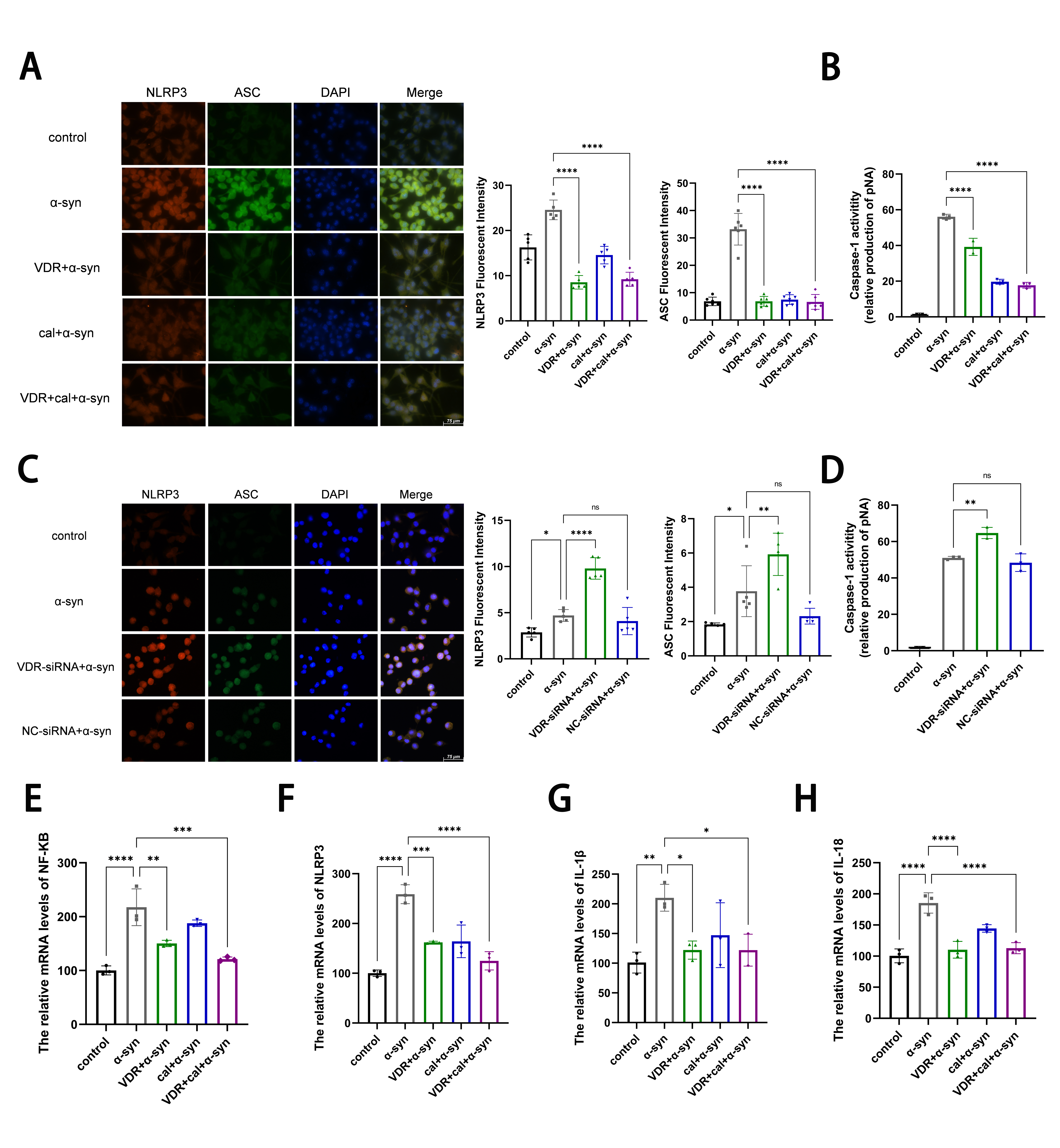

Supplement: Supplementary file 5 — Figure S4. VDR inhibits α-syn-induced inflammatory response in microglia BV2 cell line. [file 41420_2024_1912_MOESM5_ESM.jpg]

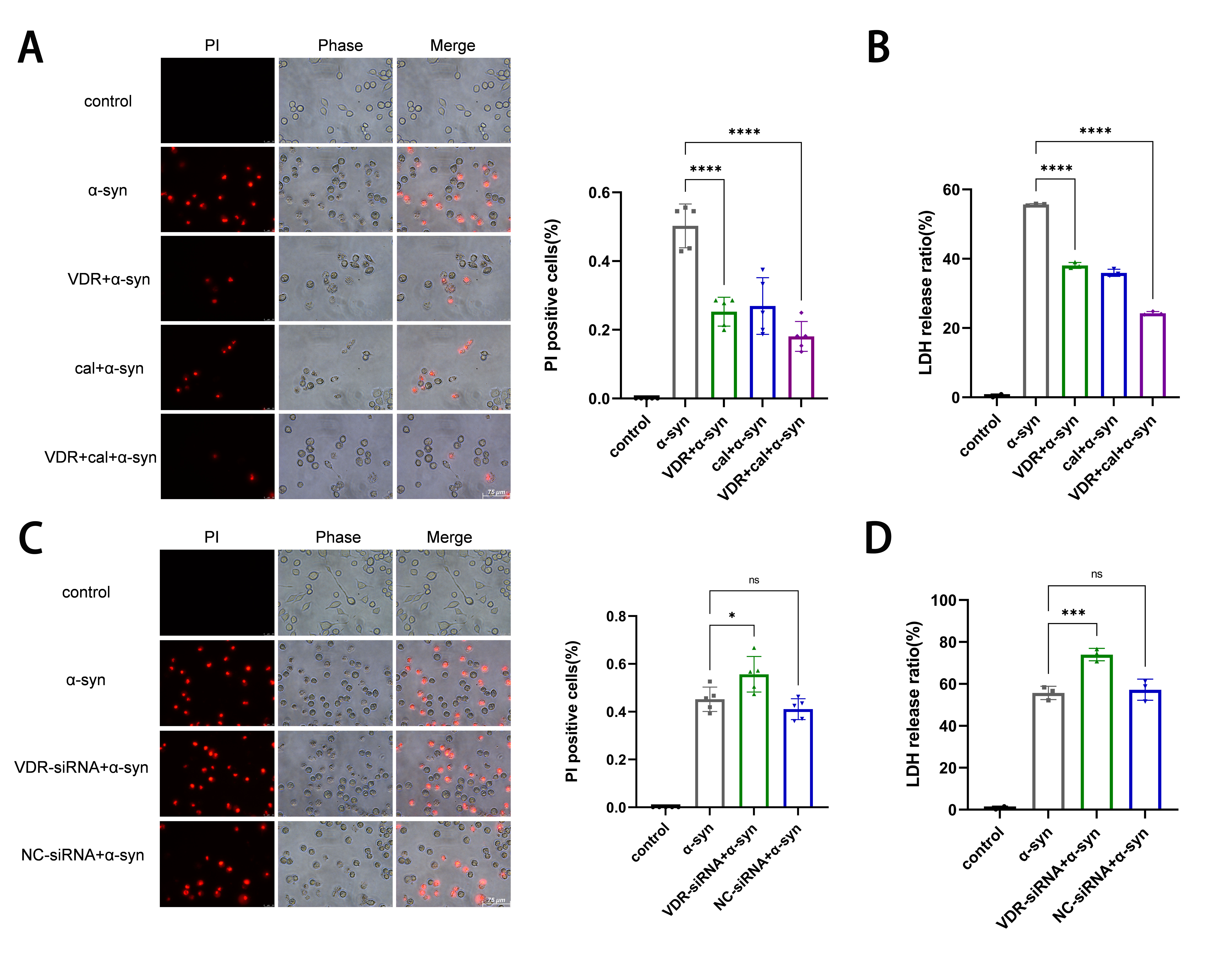

Supplement: Supplementary file 6 — Figure S5. VDR inhibits α-syn-induced microglial permeabilization in BV2 cell line. [file 41420_2024_1912_MOESM6_ESM.jpg]

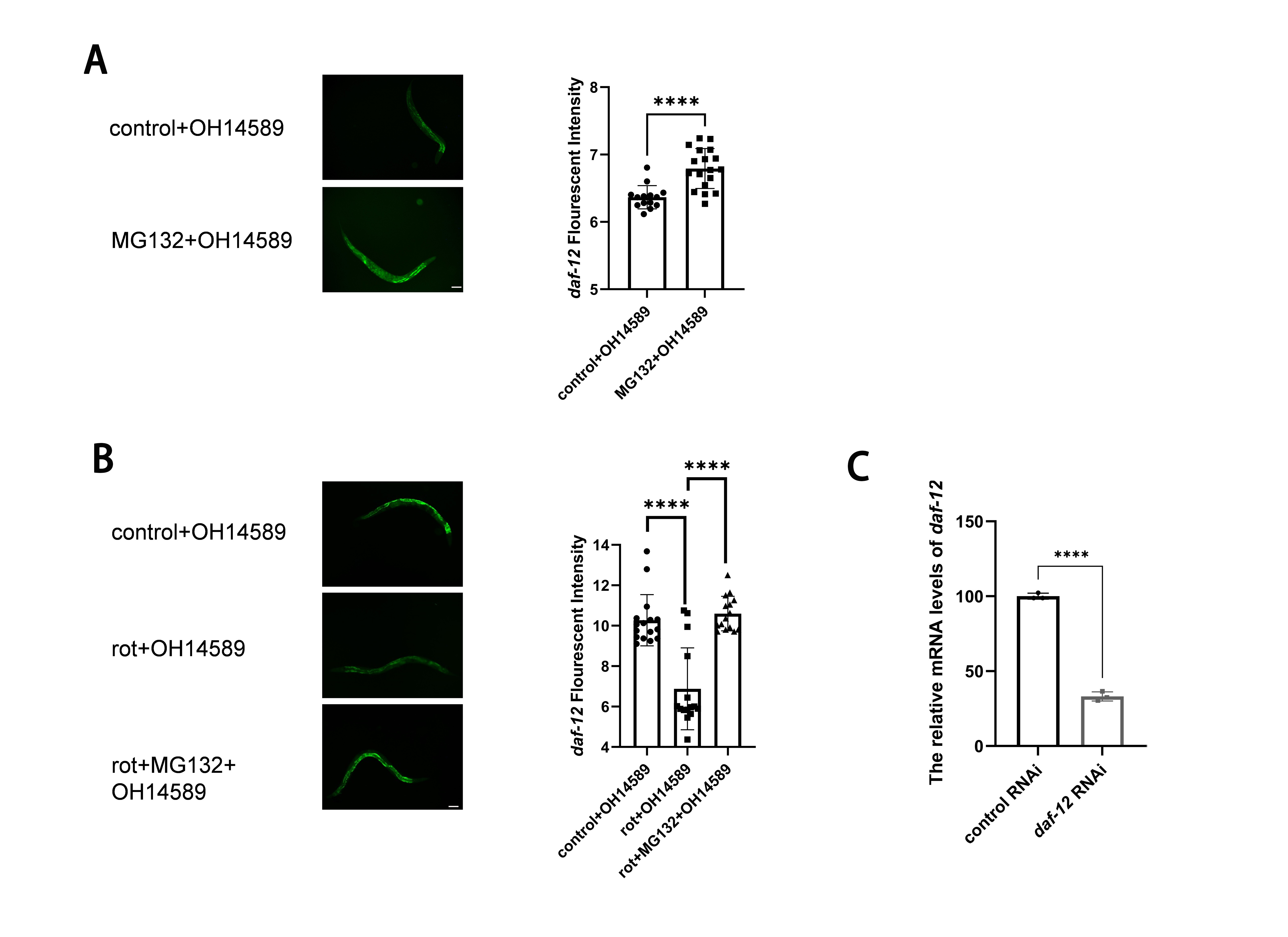

Supplement: Supplementary file 7 — Figure S6. daf-12 under ubiquitinated regulation in C. elegans. [file 41420_2024_1912_MOESM7_ESM.jpg]

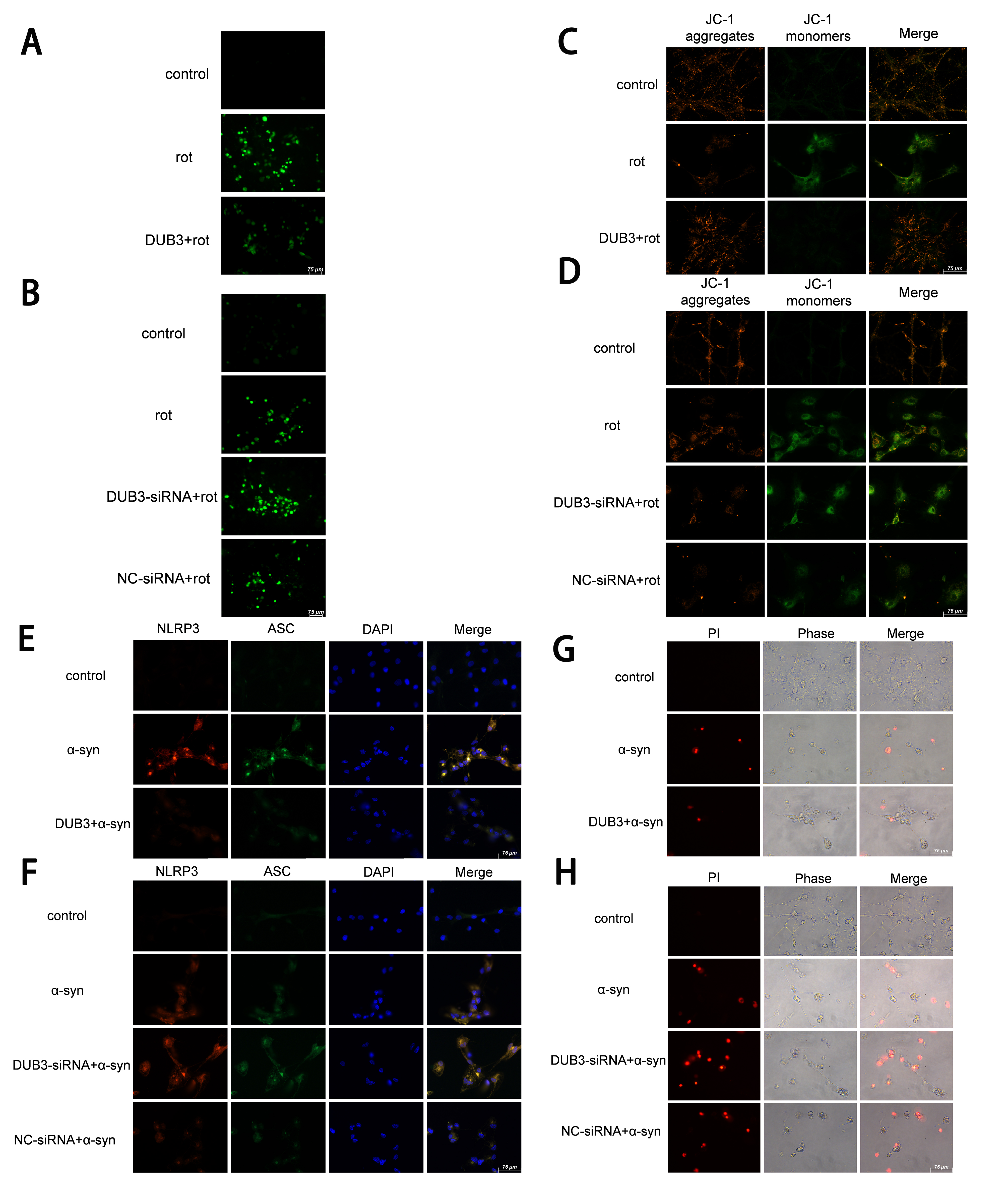

Supplement: Supplementary file 8 — Figure S7. DUB3 plays protective roles in primary neurons and microglia. [file 41420_2024_1912_MOESM8_ESM.jpg]
