# Supplementary material for: VDR and deubiquitination control neuronal oxidative stress and microglial inflammation in Parkinson’s disease
Source: Cell Death Discov. 2024 Mar 21;10:150. doi: 10.1038/s41420-024-01912-9 (PMC10957901; doi:10.1038/s41420-024-01912-9)
Supplement: Supplementary file 9 — Figure S8. Full-length uncropped original western blots [file 41420_2024_1912_MOESM9_ESM.pdf]

Figure S8. Full length uncropped original western blots

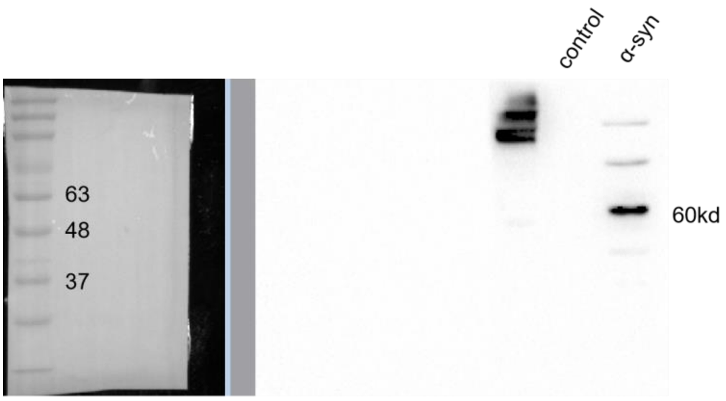

Corresponding to Fig 2A. WB: α-synuclein aggregates-60kd. GoldBand Plus 3-color Regular Range Protein Marker (8-180 kDa) was purchased from Yeasen (Shanghai, China). Synuclein alpha Ab was purchased from Affinity Biosciences (Cincinnati, USA).

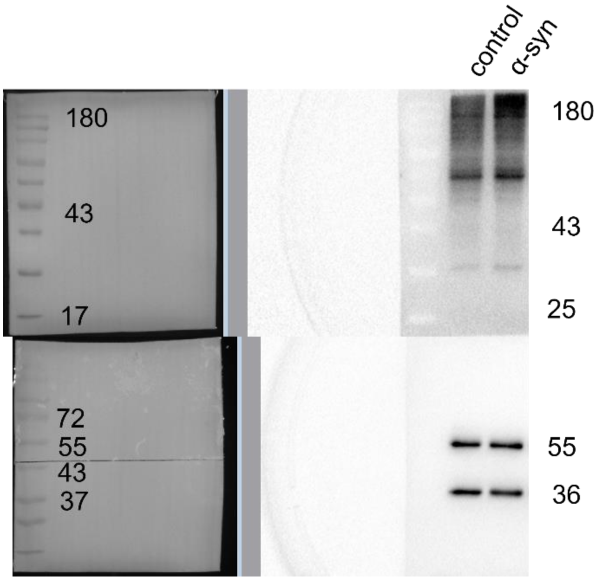

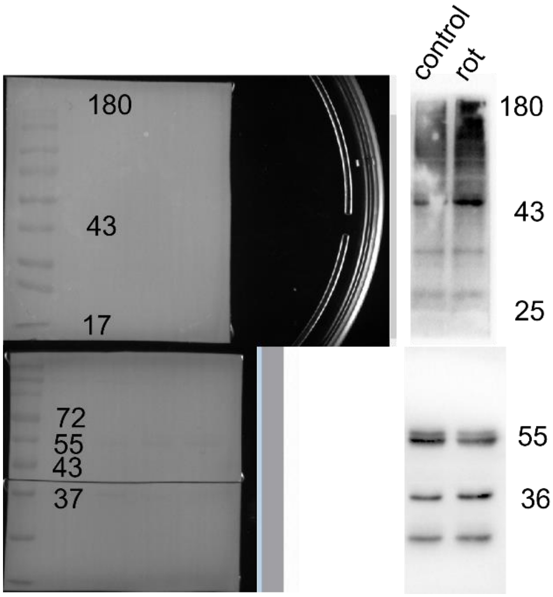

Corresponding to Fig 6A. WB: Ubiquitin, WB: VDR-55kd

GoldBand Plus 3-color Regular Range Protein Marker (8-180 kDa) was purchased from Yeasen (Shanghai, China). Purified anti-Ubiquitin Antibody was purchased from Biolegend (San Diego, USA). Vitamin D Receptor Antibody was purchased from Affinity Biosciences (Cincinnati, USA).

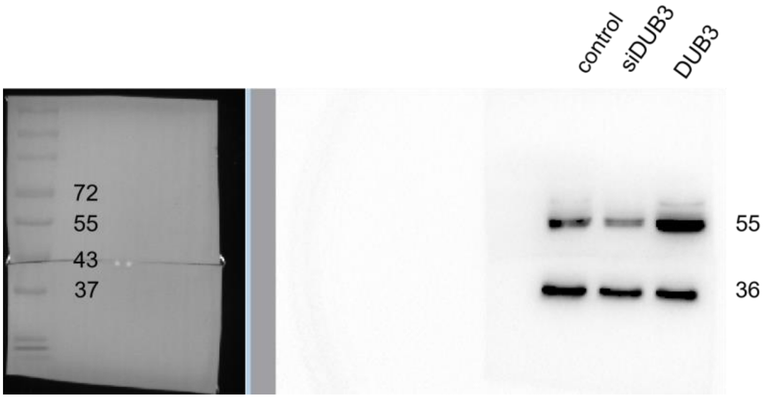

Corresponding to Fig 6B. WB: VDR-55kd

GoldBand Plus 3-color Regular Range Protein Marker (8-180 kDa) was purchased from Yeasen (Shanghai, China). Vitamin D Receptor Antibody was purchased from Affinity Biosciences (Cincinnati, USA).

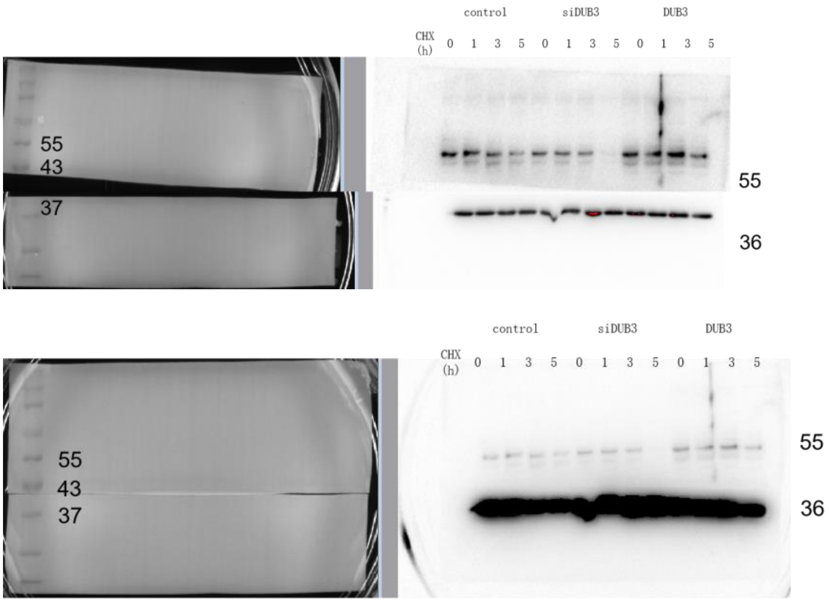

Corresponding to Fig 6D. WB: VDR-55kd

GoldBand Plus 3-color Regular Range Protein Marker (8-180 kDa) was purchased from Yeasen (Shanghai, China). Vitamin D Receptor Antibody was purchased from Affinity Biosciences (Cincinnati, USA).

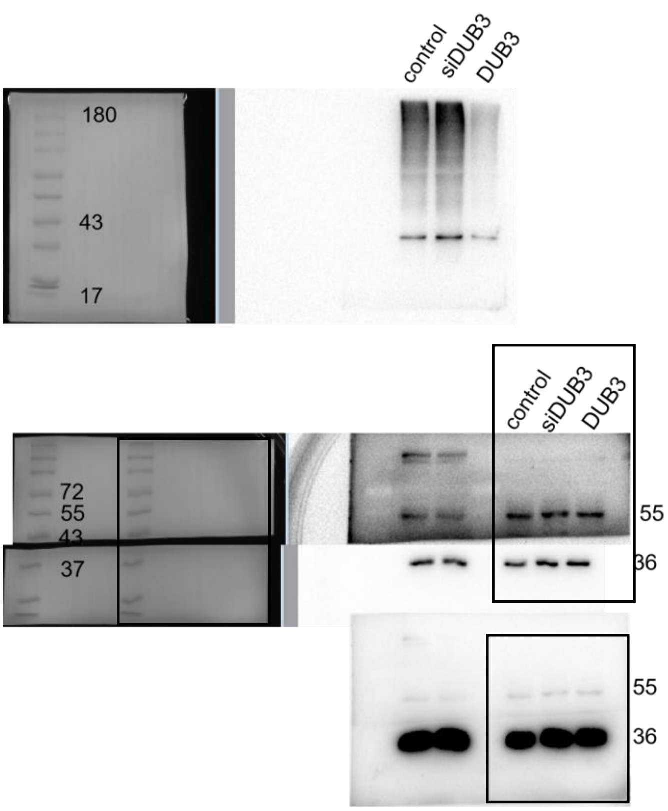

Corresponding to Fig 6E. WB: ubiquitin, VDR-55kd

GoldBand Plus 3-color Regular Range Protein Marker (8-180 kDa) was purchased from Yeasen (Shanghai, China). Purified anti-Ubiquitin Antibody was purchased from Biolegend (San Diego, USA). Vitamin D Receptor Antibody was purchased from Affinity Biosciences (Cincinnati, USA).

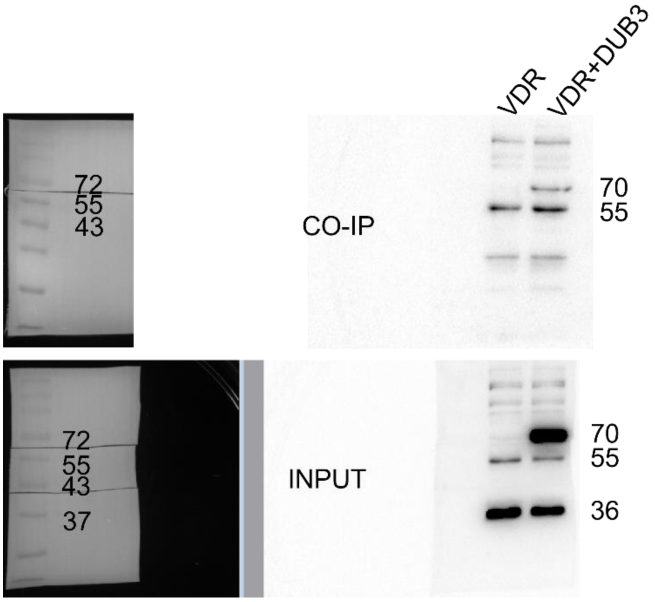

Corresponding to Fig 6F. WB: Myc-DUB3 70kd, Flag-VDR 55kd

GoldBand Plus 3-color Regular Range Protein Marker (8-180 kDa) was purchased from Yeasen (Shanghai, China). ColorMixed Protein Marker (11-180 KD) was purchased from Solarbio (Beijing, China). MYC Tag Polyclonal antibody was purchased from Proteintech (Chicago, USA). Flag Tag Antibody was purchased from Affinity Biosciences (Cincinnati, USA).

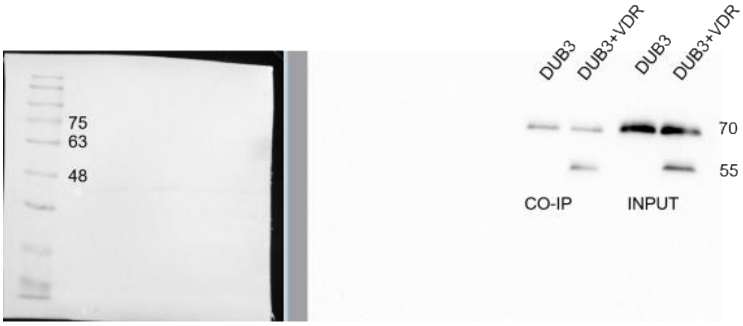

Corresponding to Fig 6G. WB: Myc-DUB3 70kd, Flag-VDR 55kd

GoldBand Plus 3-color Regular Range Protein Marker (8-180 kDa) was purchased from Yeasen (Shanghai, China). ColorMixed Protein Marker (11-180 KD) was purchased from Solarbio (Beijing, China). MYC Tag Polyclonal antibody was purchased from Proteintech (Chicago, USA). Flag Tag Antibody was purchased from Affinity Biosciences (Cincinnati, USA).

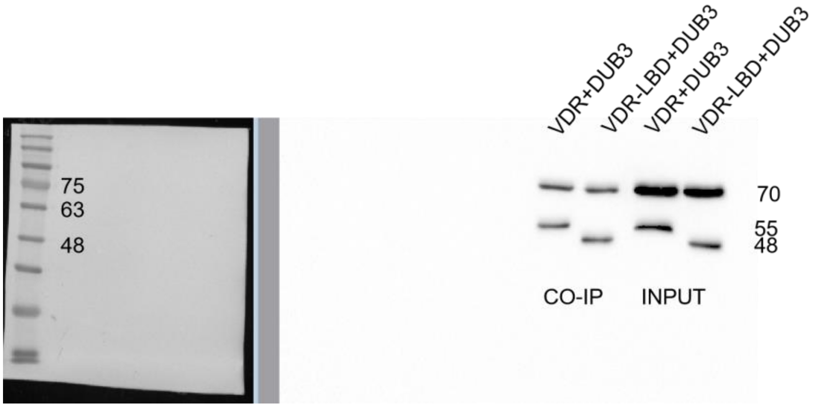

Corresponding to Fig 6I. WB: Myc-70kd, Flag-VDR 55kd, VDR-LBD 48kd

ColorMixed Protein Marker (11-180 KD) was purchased from Solarbio (Beijing, China). MYC Tag Polyclonal antibody was purchased from Proteintech (Chicago, USA). Flag Tag Antibody was purchased from Affinity Biosciences (Cincinnati, USA).

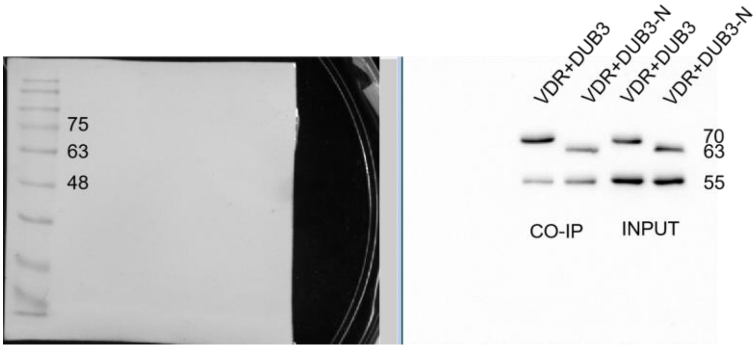

Corresponding to Fig 6J. WB: Myc-DUB3 70kd, DUB3-N 63kd, Flag-VDR 55kd

ColorMixed Protein Marker (11-180 KD) was purchased from Solarbio (Beijing, China). MYC Tag Polyclonal antibody was purchased from Proteintech (Chicago, USA). Flag Tag Antibody was purchased from Affinity Biosciences (Cincinnati, USA).

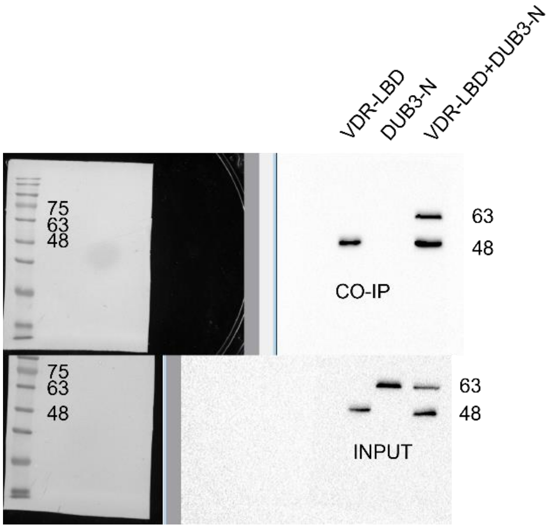

Corresponding to Fig 6K. WB: Myc-DUB3-N 63kd, Flag-VDR-LBD 48kd

ColorMixed Protein Marker (11-180 KD) was purchased from Solarbio (Beijing, China). MYC Tag Polyclonal antibody was purchased from Proteintech (Chicago, USA). Flag Tag Antibody was purchased from Affinity Biosciences (Cincinnati, USA).

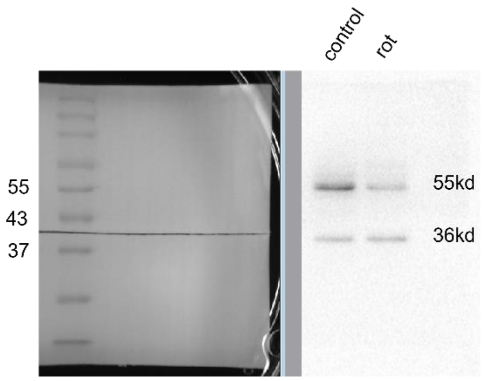

Corresponding to Fig S1A. WB: VDR-55kd

GoldBand Plus 3-color Regular Range Protein Marker (8-180 kDa) was purchased from Yeasen (Shanghai, China). ColorMixed Protein Marker (11-180 KD) was purchased from Solarbio (Beijing, China). Vitamin D Receptor Antibody was purchased from Affinity Biosciences (Cincinnati, USA).

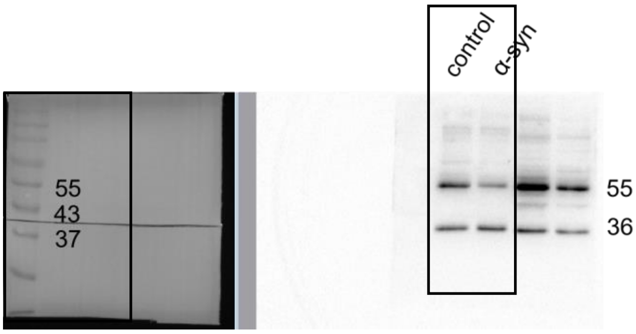

Corresponding to Fig S1B. WB: VDR-55kd

GoldBand Plus 3-color Regular Range Protein Marker (8-180 kDa) was purchased from Yeasen (Shanghai, China). ColorMixed Protein Marker (11-180 KD) was purchased from Solarbio (Beijing, China). Vitamin D Receptor Antibody was purchased from Affinity Biosciences (Cincinnati, USA).
